# Can Silver(I) Act as a Hydrogen-Bond Acceptor? Spectroscopic and Computational Exploration of the Ag···H^+^ Bonds in the Gas Phase and in Solvent

**DOI:** 10.1021/acs.inorgchem.5c03415

**Published:** 2025-12-08

**Authors:** Erik Andris, Michal Straka, Martin Dračínský, Qin Yang, Jana Roithová, Lubomír Rulíšek

**Affiliations:** † 89220 Institute of Organic Chemistry and Biochemistry of the Czech Academy of Sciences, Flemingovo náměstí 2, 16610 Praha 6, Czech Republic; ‡ Institute for Molecules and Materials, 6029 Radboud University, Heyendaalseweg 135, 6525 AJ Nijmegen, The Netherlands

## Abstract

Recent experiments highlighted the significance of Au­(I)···H interactions in the structures of Au­(I)-containing materials as well as in catalysis. However, they also raised the important question of whether similar interactions could be observed for Ag­(I) and Cu­(I) analogues of Au­(I). Herein, we present experimental and computational evidence for the formation of hydrogen bonds between the Ag­(I) center and the dimethylammonium group of protonated bis­(1-adamantyl)­(2-(dimethylamino)­phenyl)­phosphine ligand (**1**). We support this in the gas phase by employing infrared (IR) photodissociation spectroscopy correlated with quantum chemical (QC) computations. For the Ag­(I) complex (**1AgCl**_**2**_ **H**) in solution, we show by correlating the nuclear magnetic resonance data and QC calculations that the formation of the Ag­(I)···H bond competes with the formation of the Cl···H bond depending on the polarity of the environment. We evaluated interaction energies between the dimethylammonium group and the central metal across the coinage metal series (Au/Ag/Cu) using the relativistic CCSD­(T) method. Furthermore, employing wave function analysis, we gained qualitative insight into Au/Ag/Cu···H bonding at the electronic level. The definition of Ag­(I)···H interactions thus extends horizons in silver­(I) chemistry.

## Graphical abstract

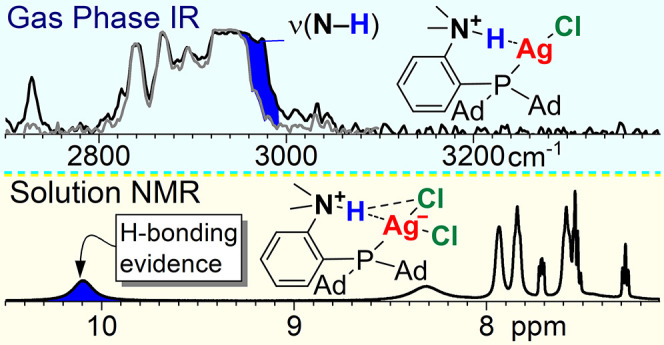

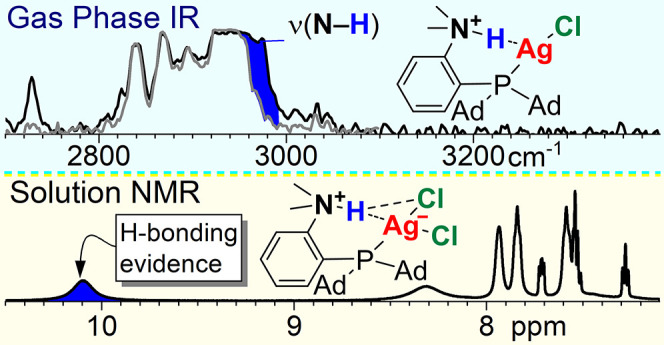

## Introduction

Hydrogen bonding to Au­(I) represents an important concept in chemistry nowadays. Yet, its existence has been a matter of controversial discussions^1,2^ and theoretical investigations^3,4^ until recently. In 2019, two independent studies provided unambiguous evidence of Au­(I)···H bonds.^5,6^ Rigoulet et al. showed that such an interaction exists in the liquid phase, via ^1^H nuclear magnetic resonance (NMR) shifts of the interacting hydrogen in [**1AuClH** ^**+**^]·OTf^–^ (Figure 1, left) and via short Au···HN^+^ distances in X-ray crystallography. Simultaneously, we presented evidence of gas-phase Au­(I)···HN^+^ bonding in **2AuClH** ^**+**^ (Figure 1, right) based on a peculiar, strongly anharmonic infrared (IR) absorption band of the NH vibration in an IR photodissociation (IRPD) spectrum. Our computational analyses^6^ revealed strong coupling of the NH vibration to low-frequency modes connected through Au­(I)···HN^+^ bonding.

**Figure 1:**
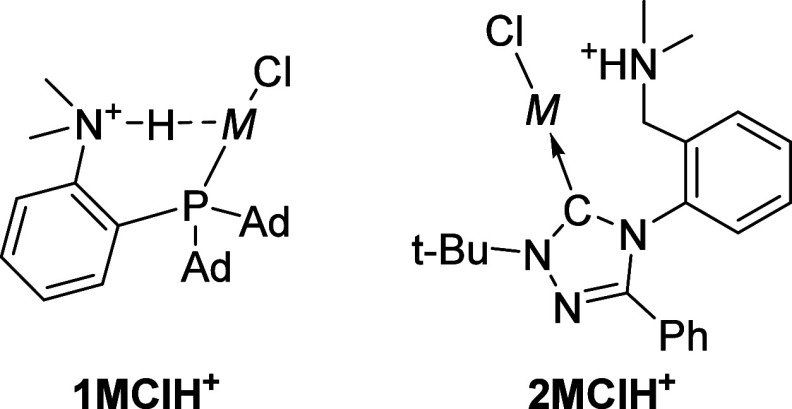
Structures of the discussed complexes featuring M···HN^+^ interactions. M = Cu, Ag, Au.

It was soon realized that Au­(I)···H bonding is responsible for many remarkable phenomena. In general, it has a stabilizing effect in various gold­(I)-containing structures. It plays an essential role in gold catalysis and C–H bond activation,^7–9^ the properties and stability of thermally activated delayed fluorescence OLED materials,^10,11^ reactions on surfaces,^12^ and the stabilization of gold nanoparticles.^13^

In light of the importance of Au···H bonding, we have recently attempted to answer the question of whether analogous Ag­(I)···H and Cu­(I)···H interactions exist.^14^ Based on the experimental and computational data obtained for the **2AgClH** ^**+**^ and **2CuClH** ^**+**^ complexes (see Figure 1), we concluded that, at least in these particular complexes, the metal–hydrogen attraction is not as favorable as in the gold­(I) counterpart, **2AuClH** ^**+**^. Still, after computational screening of about 30 different ligands, we identified several systems that could feature Ag­(I)···H or Cu­(I)···H interactions. We postulated that the most promising candidate is **1AgClH** ^**+**^, an analogue of the aforementioned **1AuClH** ^**+**^ observed in the liquid phase by the Bourissou group.^5^ Meanwhile, short M­(I)···H–C (M = Cu, Ag, Au) contacts^15^ were also studied. Such interactions are expected to be electrostatically repulsive because they are formed between positively charged atoms in M­(I)^δ+^ and H^δ+^–C fragments. Therefore, the authors in ref 16 described them as “contra-electrostatic” hydrogen bonding. They attribute their existence to stabilizing orbital (charge transfer) interactions. The presence of Cu­(I)···H interactions was also reported in the Cu­(I) bis­(benzimidazole-2-chalcogenone) complex^17^ as “intramolecular proximity-enforced Cu···H–C­(sp^3^) hydrogen bonds”. Analysis of Ag­(III)···H–C interactions in tetra­(thiocyanato)­corrolato-Ag­(III) complexes revealed that these interactions “cannot be categorically called either hydrogen bonding or agostic interactions”.^18^ All these important areas of hydrogen bonding to Group 11 metals have been recently reviewed by Sorroche et al.^19^

In this work, we present experimental and computational evidence for the existence of intramolecular Ag­(I)···HN^+^ bonding interactions. We demonstrate this for **1AgClH/D** ^**+**^ (Figure 1, left) in the gas phase by employing IRPD spectroscopy correlated with quantum chemical (anharmonic) frequency calculations. In addition, we provide experimental evidence for Ag­(I)···HN^+^ bonding interactions in the **1AgClH** ^**+**^ analogue in solution, the **1AgCl**_**2**_ **H** system, by employing NMR spectroscopy. The structural assignment is based on the correlation of experimentally determined NMR parameters with their theoretically computed counterparts. We also present the gas-phase data for the **1CuIH/D** ^**+**^ complex, including a preliminary analysis. Furthermore, we attempt to quantitatively define the strength of the M­(I)···HN^+^ interaction (M = Au, Ag, Cu) by artificially dissecting the complex into two interacting moieties (in silico). To this aim, we carried out benchmark-quality CCSD­(T)/CBS (coupled cluster with singles, doubles, and noniterative triples in the complete basis set limit) calculations, explicitly corrected for two-component relativistic effects (via X2*C* calculations). These calculations are complemented by quantum theory of atoms in molecules (QTAIM) and EDA chemical bonding analyses to understand the size and nature of Ag­(I)···HN^+^ and Cu­(I)···HN^+^ interactions.

## Methods

### Synthesis

Ligand **1** was purchased from Sigma-Aldrich. Complex **1AgCl** was prepared by mixing 42.1 mg of **1** with 14.3 mg of AgCl in 2 mL of dichloromethane (DCM) in an ultrasound bath at room temperature until everything dissolved. This took about 1 h, during which the temperature of the bath increased to approximately 40 °C due to ultrasound operation. After everything was dissolved, the colorless solution was dried and the product (white powder) was used directly for further characterization and experiments, as detailed below. No uncommon hazards are noted.

### Electrospray Ionization Mass Spectrometry

Electrospray ionization mass spectrometry experiments were conducted using a TSQ 7000 triple quadrupole mass spectrometer (Finnigan), equipped with an electrospray ion source. 50 μL of a 1 mM solution of **1AgCl** in DCM was dissolved in 500 μL of MeOH and sprayed. Illustrative ionization conditions: no auxiliary gas or sheath gas flow, small vial with glacial AcOH present in the ion source, 6.5 kV, 120 °C, −25 V capillary voltage, 137 V tube lens voltage, −1.3 V transfer quadrupole offset, 1.5 V L11 voltage. Complex **1CuI** was prepared similarly to **1AgCl** by mixing 9 mg of CuI with 20 mg of **1** in 1 mL of DCM and putting the whole mixture to ultrasound (no efforts were made to exclude oxygen). 10 μL of the resulting solution was then added into 2 mL of MeOH and analyzed by electrospray ionization (ESI) (example ionization conditions: 7.5 kV spray voltage, 150 °C capillary temperature, −5 V capillary voltage, 97 V tube lens voltage, 1 psi sheath gas pressure). For the isotopic labeling experiments, MeOH was substituted by MeOD and auxiliary gas (20–60 au flow) and sheath gas (8–40 psi) were used for the ionization to exclude atmospheric moisture, along with smaller tube lens voltage (87,97 V for **1CuI**). The AcOH in the ion source (used only for experiments with **1AgCl**) was unlabeled.

### IRPD Spectra

IRPD spectra were acquired on the ISORI instrument as described previously.^20,21^ Typical ionization conditions are described in the Electrospray Ionization Mass Spectrometry section.

### NMR Spectra

NMR spectra were recorded on a 500 MHz Bruker NEO NMR spectrometer (^1^H at 500 MHz, ^13^C at 126 MHz, ^31^P at 203 MHz, and ^109^Ag at 23 MHz) in CD_2_Cl_2_. The spectra were measured using a triple-resonance probe enabling mutual irradiation/detection of ^1^H, ^31^P, and ^109^Ag nuclei. Proton spectra were referenced to the solvent signal δ = 5.32 ppm. 10 mg of **1AgCl** was dissolved in DCM, and a small amount of concentrated water solution of HCl or 1 equiv of 4 M HCl in dioxane was added while cooling the solution in a dry ice/ethanol bath. The sample was then transferred to an NMR spectrometer, where it was cooled to −70 °C and later heated as described.

### Density Functional Theory Calculations of Anharmonic Frequencies

Anharmonic IR spectra were calculated with the Gaussian 16 program,^22^ using the GVPT2 approach for anharmonic calculations. B3LYP^23–26^ density functional theory (DFT) functional with D3 dispersion correction,^27,28^ 6-31++G­(d,p) basis set on C, H, N, O, P, Cl, and Cu atoms, and the def2-SVPD on Ag and I atoms were used. Anharmonic vibrational analyses were recalculated with the development version of the Gaussian program, kindly provided by Dr. Julien Bloino. For the calculation of **1CuIH** ^**+**^ anharmonic vibrational frequencies, it was necessary to use tighter convergence criteria to avoid artificial and spurious IR intensities of many bands. These were “Opt­(VeryTight), CPHF­(Grid = OneStep), Scf­(Conver = 10,xqc), Int­(SuperFineGrid)”. In order to speed up the calculations, individual displaced geometries for the numerical third and fourth derivative calculations were split into individual jobs in a quasi-parallel way; the jobs were calculated at the same time and assembled for the anharmonic analysis with the help of the “External” interface to Gaussian.

### DFT Calculations of NMR Parameters

Calculations of NMR parameters were carried out using the ADF code, version 2023.101.^29^ Following, to a great extent, our previous experience,^30^ we used PBE0-D3/def2-TZVPP/ECP­(M)/PCM geometries (optimized using the Turbomole 7.8 software^36,37^ ) as input for SO-ZORA PBE0/TZ2P-J/PCM NMR calculations.^30,31^ The COSMO solvation model using DCM as a solvent (ε_r_ = 8.93) was employed both in geometry optimizations and in NMR calculations as implemented in individual codes. The chemical shifts were obtained from calculated isotropic shielding constants σ_iso_ according to the formula δ = (σ_ref_ – σ_iso_)/(1 – σ_ref_ × 10^–6^) + δ_sec_, where PH_3_ and AgPy_2_^+^ were calculated as secondary references (σ_ref_) for ^31^P and ^109/107^Ag shifts, respectively. Tetramethylsilane was used as reference for ^1^H shifts. The results were scaled to the real reference used in the experiment (H_3_PO_4_ and AgNO_3_) by a δ_sec_ of −226 ppm for ^31^P and +350 ppm for ^109/107^Ag nuclei, respectively. The *J*-couplings were calculated at the SO-ZORA PBE0/TZ2P-J/PCM level for ^109^Ag, ^31^P, and ^1^H isotopes. Where needed, the values were averaged or recalculated for ^107^Ag *J*-couplings using a gyromagnetic ratio of 1.0878 × 10^7^ rad/Ts for ^107^Ag and 1.25 × 10^7^ rad/Ts for ^109^Ag.

### Bonding Analyses

The QTAIM analysis^32^ was performed with the AIMALL program.^33^ The EDA–NOCV analysis^34^ (energy decomposition analysis–natural orbitals for chemical valence) was performed using the implementation in the ADF program.^29^ Natural bond orbitals analysis^35^ was performed using Gaussian 16.^22^

### Coupled Cluster and X2C Calculations

For model systems, we carried out the reference (canonical) coupled cluster calculations with singles, doubles, and noniterative triples, CCSD­(T), employing Turbomole 7.8. software.^36,37^ Two large basis sets were used, aug-cc-pVTZ, and aug-cc-pVQZ,^38–41^ to allow for the complete basis set limit extrapolation (vide infra). For Ag and Au, Stuttgart–Dresden effective core potentials^42^ (*N*_core_ = 28 and 60 for Ag and Au, respectively) were used. The number of frozen orbitals in the CCSD­(T) calculations was set to 0 (which was somewhat unnecessary, but still computationally affordable; this prevented unwanted mixing of core orbitals from heavier halogens with the semicore orbitals of coinage metals within the set of frozen orbitals). To account for explicit relativistic effects beyond the effective core potential treatment (e.g., spin–orbit coupling; even though they were expected to have a very small effect on the computed interaction energies), we carried out X2C-PBE/X2C-TZVPall-2c calculations^43,44^ and compared then with the corresponding PBE/def2-TZVP­(ECP) calculations.

## Experimental Results

### Gas-Phase IRPD Characterization of **1AgClH** ^**+**^ and **1CuIH** ^**+**^

We sprayed the clear solution that formed after sonication of **1** with AgCl from MeOH and with AcOH added in the ion source since the complex decomposed when acid was added directly to the solution. Due to its corrosive properties, we did not try to protonate **1AgCl** in the ion source by HCl. The main peaks in the resulting mass spectrum (Figure S1) correspond to **1H** ^**+**^ (*m*/*z* 422) and *m*/*z* 263. The main silver species in the spectrum are at *m*/*z* 564, corresponding to **1AgClH** ^**+**^, at *m*/*z* 588 (possibly **1AgOAc** ^**+**^), and at *m*/*z* 624 (possibly **1AgCl.AcOH** ^**+**^). Collision-induced dissociation of *m*/*z* 564 with xenon at 19 eV (in the laboratory frame) revealed two main channels: loss of AgCl (*m*/*z* 422) and loss of HCl (*m*/*z* 528) (see Figure S2).

To confirm that the peak at *m*/*z* 564 corresponds to the proposed **1AgClH** ^**+**^ structure (Figure 1, left), we measured its infrared photodissociation (IRPD) spectrum using the ISORI instrument (Figure 2A, black trace). The absence of peaks between 3200 and 3800 cm^–1^ (not shown in the figure) confirms the absence of free N–H bonds. To locate the N–H stretching mode, we used deuterium labeling, exchanging the N–H group for an N–D group (Figure 2A, blue trace). We observed the disappearance of three bands: one relatively strong band at 2968 cm^–1^ (see also the spectrum measured with lower laser power—shown in gray) and two weaker bands at 2726 cm^–1^ and 2694 cm^–1^, respectively. The low frequency of the 2968 cm^–1^ band confirms the presence of a hydrogen-bonded N–H group in the molecule. Our GVPT2 calculations indicate that the 2968 cm^–1^ band likely corresponds to the stretching ν­(N–H) band (cyan peak at 2892 cm^–1^ in Figure 2B) and that the two lower-frequency peaks correspond to overtones of the two δ­(N–H) deformation bands (magenta peaks, Figure 2B), respectively. However, we must admit that an unambiguous 1:1 mapping of the GVPT2 variational vibrational states to the harmonic states is not possible.

**Figure 2:**
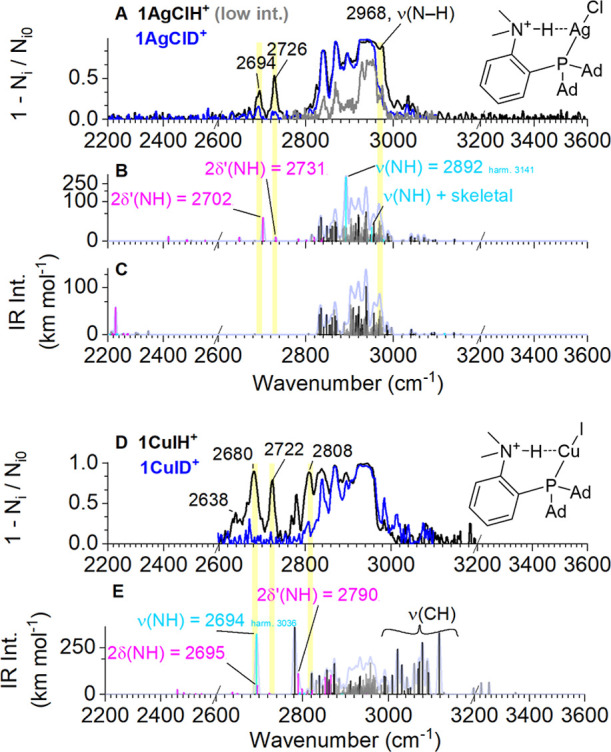
IR spectrum of **1AgClH** ^**+**^ (A, black trace) and **1CuIH** ^**+**^ (D, black trace). Blue traces in panels (A) and (D) correspond to the spectra of deuterated species. The gray trace in panel (A) corresponds to the spectrum of **1AgClH** ^**+**^ measured with less laser power (the spectrum is less saturated). Predicted anharmonic IR spectra of **1AgClH** ^**+**^ and **1AgClD** ^**+**^ (B,C) at the GVPT2 level. Predicted anharmonic IR spectrum of **1CuIH** ^**+**^ (E) at the GVPT2 level. Harmonic frequencies of ν­(NH) in **1AgClH** ^**+**^ and **1CuIH** ^**+**^ are predicted to be 3141 and 3036 cm^–1^, respectively.

For comparison, the resonance at 2968 cm^–1^ is higher than the previously measured resonance for the N–H^+^···Cl bond in **2AgClH** ^**+**^, where the maximum intensity of the band was at 2595 cm^–1^, ref 6, suggesting (expectedly) a weaker interaction in the present case. The respective vibration in **1AuClD** ^**+**^ (deuterated analogue of **1AuClH** ^**+**^, thus not directly comparable to our results) measured in solution was found at 2124 cm^–1^ (ref 5), but we were not able to measure this range of IR spectrum for the complexes studied in this work, due to technical difficulties with the OPO/OPA laser system. Likewise, the spectrum of **1AuClH** ^**+**^ has not been reported, making a direct comparison impossible.

We also attempted to measure the spectra of the **1CuIH** ^**+**^ complex and its deuterated analogue (Figure 2D). The situation was quite similar to the above-discussed spectra of **1AgClH** ^**+**^; but this time, there were 4 bands that disappeared upon deuterium labeling: 2808, 2722, and 2680 cm^–1^ and a weaker band at 2648 cm^–1^. The middle two resembled the two lower bands from the **1AgClH** ^**+**^ spectrum that we assigned as the δ­(NH) overtones. From the position of the highest band, we may speculate that the interaction of the NH group is slightly stronger than that in the silver complex. The GVPT2 calculation also predicts that the ν­(NH) band in **1CuIH** ^**+**^ should be present at 2694 cm^–1^ (Figure 2E), which is lower than the predicted value for the silver complex (2892 cm^–1^, Figure 2B). However, the predicted anharmonic intensities in the GVPT2 calculations do not accurately reproduce the experiment. For example, the C–H stretching vibrations above 3000 cm^–1^ are much stronger than in reality. We improved the intensities by running the calculations with more accurate numerical thresholds; however, we were not able to completely eliminate these artifacts. Therefore, our conclusions about the **1CuIH** ^**+**^ system based on the IRPD spectra possess greater uncertainty, compared to the **1AgClH** ^**+**^ system.

### NMR Characterization of **1AgCl**

Mixing solid AgCl with **1** in DCM in an ultrasonic bath at room temperature resulted in the dissolution of AgCl within approximately 1 h, after which a clear solution was formed. The solid obtained after evaporating DCM was characterized as **1AgCl** using NMR spectroscopy in a dichloromethane-*d*_2_ solution (Figures S3–S7). The ^31^P NMR spectrum of **1AgCl** in CD_2_Cl_2_ exhibits four peaks, centered at 39 ppm (37 ppm at −70 °C, Figure S7 and Figure 3C). Silver has two stable isotopes: ^109^Ag with a natural abundance of 48% and ^107^Ag with a natural abundance of 52%. Both have a nuclear spin quantum number of *I* = 1/2. Thus, the four lines in the ^31^P spectrum correspond to two doublets. One with a slightly higher intensity represents **1** ^**107**^ **AgCl** with a signal splitting of *J*(^107^Ag,^31^P) = 602 Hz. The second doublet represents **1** ^**109**^ **AgCl** with *J*(^109^Ag,^31^P) = 694 Hz (Figure S7). Note that the ratio of *J*-couplings corresponds to the ratio of the gyromagnetic ratios of ^107^Ag and ^109^Ag (see Methods). The structure of **1AgCl** was further confirmed by the *J*-couplings between the carbon and phosphorus nuclei, as well as between the phosphorus and silver nuclei (see Figures S6 and S7).

**Figure 3:**
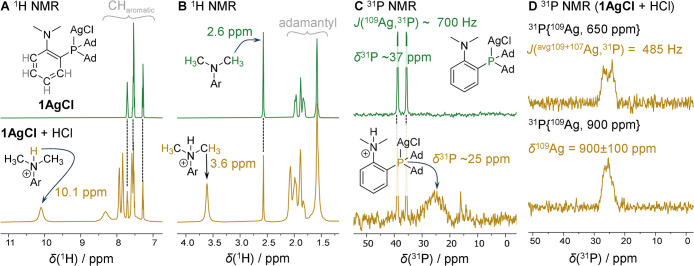
NMR spectra of **1AgCl** before (green) and after (yellow) the addition of HCl measured in CD_2_Cl_2_ at −70 °C. (A) The aromatic region of the ^1^H NMR spectrum; (B) the aliphatic region of the ^1^H NMR spectrum; (C) ^31^P spectrum. The addition of HCl led to the formation of a mixture of neutral and protonated **1AgCl** in the ratio 1:2.4. (D) The ^31^P NMR spectra of **1AgCl** after the addition of HCl measured in CD_2_Cl_2_ at −60 °C using the ^109^Ag decoupling at 650 ppm (top) and at 900 ppm (bottom). The disappearance of the doublet at 900 ppm ^109^Ag decoupling suggests that the ^109^Ag shift of the protonated compound is 900 ± 100 ppm.

It should be mentioned that both silver isotopes have very low sensitivity in NMR spectroscopy, and Ag spectra can only be obtained for highly concentrated solutions, which were not achievable for this sample. We were, however, able to probe the ^109^Ag NMR shift indirectly by measuring ^109^Ag-decoupled ^31^P NMR spectra with a varying ^109^Ag decoupling frequency. When the irradiation frequency matches the resonance frequency of ^109^Ag in the sample, the ^31^P­(^109^Ag) doublet collapses into a singlet while the ^31^P­(^107^Ag) doublet remains unchanged. We estimated the selectivity of the ^109^Ag decoupling to be approximately ±50 ppm in the case of **1AgCl**. The greatest decoupling was observed at δ­(^109^Ag) = 650 ppm (Figure S7B). In this way, we established the ^109^Ag chemical shift in **1AgCl** to be 650 ± 50 ppm with respect to the AgNO_3_/H_2_O reference.

### NMR Characterization of the Protonation Product of **1AgCl**

Encouraged by the observation of the **1AgClH** ^**+**^ cation in the gas phase, we investigated the potential formation of **1AgClH** ^**+**^ in solution. However, since we observed only the protonated ligand **1H** ^**+**^ in the ESI-MS spectrum of the **1AgCl**/HCl mixture, we suspected that **1AgClH** ^**+**^, if it exists in solution at all, would have low stability. Therefore, we began our investigation by adding small amounts of acid to the DCM solution of **1AgCl** at −70 °C.

Our attempts to protonate **1AgCl** with triflic acid in analogy to ref 5 were not successful. We observed the formation of **1H** ^**+**^, in which silver was no longer attached to the ligand. The ^31^P NMR spectra showed a singlet signal at 16.4 ppm (Figure S8), while the splitting of the signal due to *J*-coupling with both silver isotopes is lost. Furthermore, a new doublet appeared in the proton spectrum at 6.64 ppm with a large ^1^H–^31^P coupling of 481 Hz, providing evidence that the proton is directly attached to the phosphorus atom (Figure S9). These results agree with those previously reported by Rigoulet et al.,^5^ δ­(^31^P) = 17.5 ppm and *J*(^31^P–^1^H) = 485 Hz. It is quite clear that protonation by HOTf leads to the formation of **1H** ^**+**^ and the precipitation of AgCl, which we observed as a white precipitate after the experiment. We also attempted to protonate the silver complex by acetic acid, but this did not result in the protonation of the complex.

The situation was quite different upon addition of HCl (either concentrated HCl solution in water or 4 M HCl in dioxane) to a solution of **1AgCl** at −70 °C. A new broad signal in the 22–30 ppm range of the ^31^P NMR spectrum (Figure 3C) was recorded. Additionally, the ^1^H NMR spectrum showed a new signal at 10.1 ppm (Figure 3A). This signal was assigned to an N-bonded hydrogen atom based on a ^1^H,^15^N-HSQC experiment (Figure S11). Note that **1AuClH** ^**+**^, reported by Rigoulet et al., featured a ^1^H signal at 10.9 ppm.^5^ The ^1^H chemical shift of the dimethylamino group also significantly changes from 2.6 to 3.6 ppm in **1AgClH** ^**+**^, also indicating that the proton is attached to the dimethylamino group (Figure 3B). In addition, we observed downfield NMR shifts of the aromatic C–H protons by ca. 0.5 ppm on average with respect to their counterparts in **1AgCl**.

After the sample was heated to −60 °C and then to −50 °C, the broad signal at 22–30 ppm in the ^31^P spectrum changed into a broad doublet (Figure 3D). The splitting of the signal is caused by its *J*-coupling with silver, which was confirmed by ^109^Ag decoupling. We observed partial decoupling of this signal with the ^109^Ag irradiation frequency at 1000 ppm, the strongest decoupling occurred at 900 ppm, and no decoupling was observed at 800 ppm, allowing us to assign the ^31^P signal to a silver-containing species with a ^109^Ag shift of 900 ± 100 ppm (Figure S13; these experiments were done with HCl in dioxane (instead of aqueous conc. HCl), but we observed the same species decoupling at the same ^109^Ag shift in aqueous HCl in Figure 3D). The average ^107/109^Ag,^31^P *J*-coupling is 485 Hz (we could not discern the signals corresponding to the two isotopomers as the peaks are too broad; this value corresponds to 451 and 519 Hz for ^107^Ag/^109^Ag,^31^P *J*-coupling, respectively). Upon further heating, the complex slowly decomposes, even though the overall spectra seemed a little cleaner and the protonated complex more stable, when we used aqueous HCl instead of HCl in dioxane. This suggested that the presence of water might have a stabilizing effect on the formed complex (see below).

To elucidate the structure of the product(s) in solution further, we performed a series of calculations of NMR parameters at the SO-ZORA PBE0/TZ2P-J/COSMO­(DMC) level (see Methods for details). To assess the accuracy and reliability of these calculations, we first calculated the NMR parameters for the **1AgCl** system. The calculated δ­(^31^P) = 41.8 overestimates the experimental value of 37 ppm by ∼5 ppm, which is slightly higher than the typical error of 2–3 ppm, based on our previous experience.^45^ On the other hand, the calculated *J*(^109^Ag,^31^P) = −704 Hz for **1AgCl** (Figure 4A) is in very good agreement with the experimental value of 694 ppm (note that only the absolute value was obtained in the experiment). Additionally, the calculated δ­(^109^Ag) of ∼666 ppm overlaps well with the observed value of δ­(^109^Ag) ∼ 650 ± 50 ppm. This provides calibration data for the expected accuracy of our Ag chemical shift calculations. Last but not least, the average δ­(^1^H) chemical shift on the methyls of the dimethylamine group is well reproduced (calculated 2.7 ppm vs measured 2.6 ppm).

**Figure 4:**
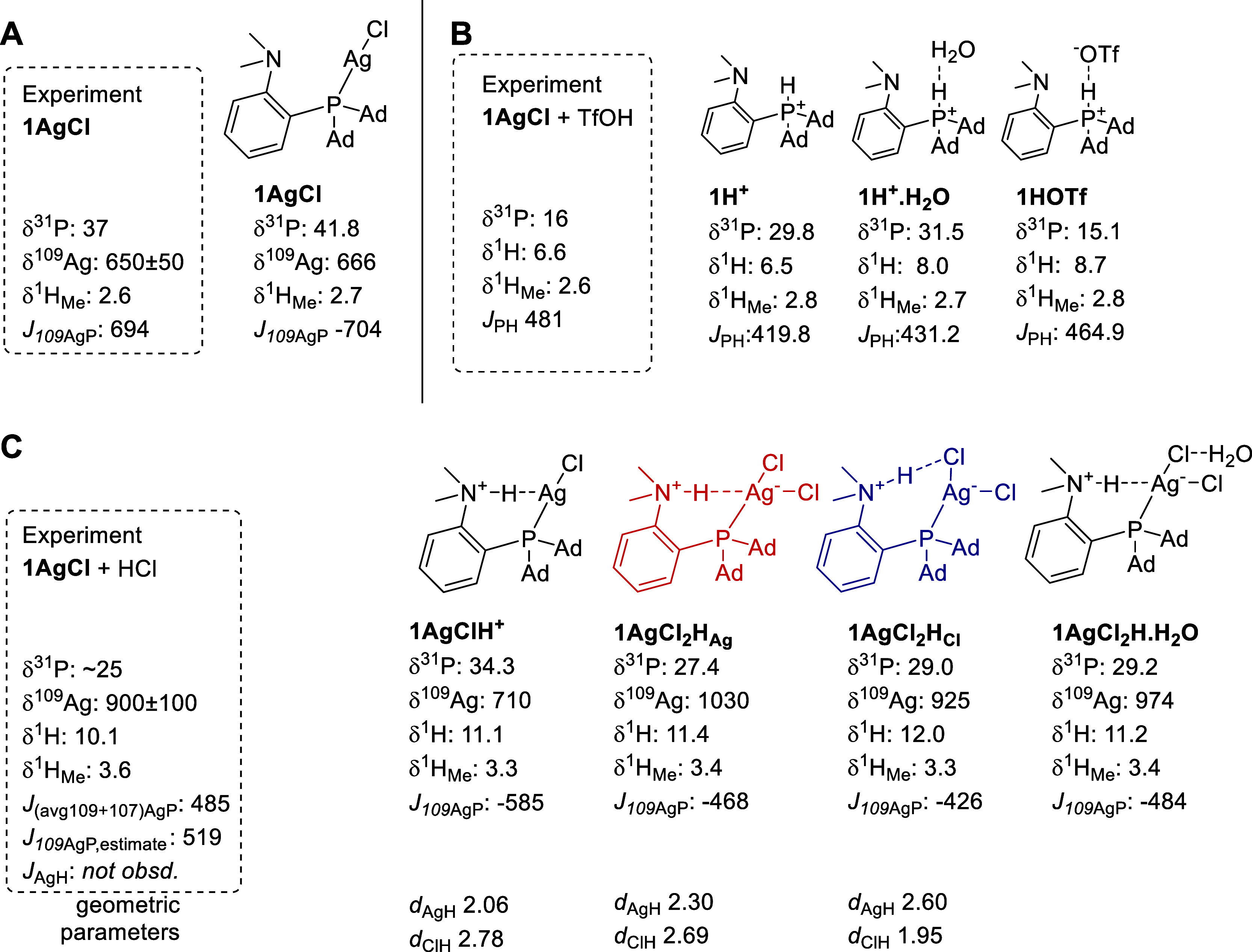
Computed NMR parameters for multiple structural arrangements relevant to the present study. (A) Neutral complex, (B) protonated ligand, and (C) various protonated forms of the silver complex. Additional structures can be found in Figure S14. The chemical shifts are reported in ppm, whereas the *J* coupling constants are given in Hz. The experimental *J* couplings are only available and given here in absolute values.

The predicted NMR parameters for the bare **1H** ^**+**^ system (Figure 4B): δ­(^1^H) = 6.50 ppm, δ­(^31^P) = 29.8 ppm, *J*(^1^H–^31^P) = 419.8 Hz are in good agreement with the experiment, except for the δ­(^31^P) where the experimental value is 16.4 ppm. A closer investigation reveals that NMR parameters in **1H** ^**+**^ are rather sensitive to the presence of a H-bond acceptor of the P–H group, such as a water molecule or a triflate counterion (cf. **1H** ^**+**^ **.H**_**2**_ **O** or **1HOTf** in Figure 4B). This effect is usually not well described by an implicit solvation model. Indeed, computed NMR parameters for the **1HOTf** system, δ­(^31^P) and *J*(^1^H–^31^P), are much closer to the experimental values (Figure 4B). At the same time, we observed an overestimation of δ­(^1^H) = 8.71 ppm vs the experimental value of 6.6 ppm. We speculate that dynamical averaging would improve the overall theoretical description of the **1H** ^**+**^ NMR parameters. However, this is beyond the scope of this work.

Now, let us consider an experiment in which **1AgCl** was mixed with HCl (Figure 4C). Calculations suggest that protonated **1AgClH** ^**+**^ is actually not present in the solution, as there are large differences between the predicted and experimental ^109^Ag NMR shifts (710 ppm vs 900 ± 100 ppm, respectively). Additionally, the computationally predicted δ­(^31^P) value of 34.3 ppm falls outside the error range compared with the measured δ­(^31^P) value of ∼25 ppm.

Much better agreement between the predicted NMR data and their experimental counterparts is observed for the neutral **1AgCl**_**2**_ **H** system. Interestingly, the PBE0/def2TZVPP/PCM minimization of **1AgCl**_**2**_ **H** revealed two almost isoenergetic minima (conformers), one with an N–H bond pointing toward the silver (Ag···HN bonding) and one with an N–H bond pointing toward the chloride (Cl···HN bonding). These are denoted as **1AgCl**_**2**_ **H**_**Ag**_ and **1AgCl**_**2**_ **H**_**Cl**_, respectively (Figure 4C). Notably, these two conformers are very close in energy; this fact is reproduced at various computational levels (Table S1, Figure 5). Despite some differences in their calculated NMR properties, we cannot unambiguously discern between the two conformers. The agreement of the Ag NMR chemical shift is better for **1AgCl**_**2**_ **H**_**Cl**_ (925 ppm compares better with the experimental value of 900 ± 100 ppm than the computed values of 1030 ppm for **1AgCl**_**2**_ **H**_**Ag**_), whereas the δ­(^31^P) and *J*(^109^Ag,^31^P) values are in better agreement for **1AgCl**_**2**_ **H**_**Ag**_ (Figure 4C). The addition of an explicit water molecule to **1AgCl**_**2**_ **H** (resulting in **1AgCl**_**2**_ **H·H**_**2**_ **O**, a structure that might be relevant for the experiments, where we added HCl in the form of concentrated solution in water; cf. Figure 4C) leads to stabilization of the **1AgCl**_**2**_ **H**_**Ag**_-like structure (Figure 4C), and the same is true if we increase implicit solvent polarity (Table S1). Based on additional calculations of various structural alternatives and model systems, depicted in Figure S14, we can conclude that the system present in solution is indeed **1AgCl**_**2**_ **H**, but we cannot distinguish between its two equilibrium geometries that are almost isoenergetic. They can most likely be distinguished by the *J*(^109^Ag,^1^H) coupling of the silver atom with the dimethylammonium hydrogen, computed at 2.1 Hz for **1AgCl**_**2**_ **H**_**Ag**_ and 0.1 Hz for **1AgCl**_**2**_ **H**_**Cl**_. However, the peak width of the hydrogen signal at 10 ppm is too broad (40 Hz) to distinguish between these possibilities.

**Figure 5:**
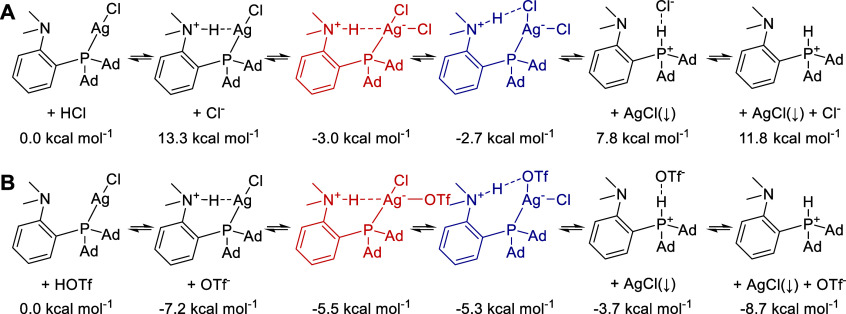
Gibbs free energies (Δ*G* ^298 K,1 M^) for the protonation of **1AgCl** by (A) HCl and (B) HOTf calculated at the PBE0-D3BJ/def2-TZVPP//PBE0-D3BJ/BS1 (BS1 = dgauss-dzvp on atoms with *Z* = 1–18, def2-TZVP on other atoms) level using ORCA 6.0.1 ^101^ with Δ*G*_solv_ added from the COSMO-RS solvation model^102^ (BP_TZVPD_FINE_23.ctd parameters; calculated at the BP86 ^103,104^ /def2-TZVPD level with FINE cavity^105^ on the PBE0 geometries) in dichloromethane, calculated using Turbomole 7.8 and COSMOTherm 23 (Biovia) programs. The energy of solid AgCl to use with the DFT energies was calculated as Δ*G*(AgCl,DFT,s) = Δ*G*(Ag^+^,DFT,gas) + Δ*G*(Cl^–^,DFT,gas) – (Δ_f_ *G*(Ag­(g))+IE­(Ag)) – (Δ_f_ *G*(Cl­(g))–EA­(Cl)) + Δ_f_ *G*(AgCl(s)), where EA is the electron affinity. The ionization energy (IE) of the Ag atom was taken from ref 46, whereas the Gibbs free energies of formation (Δ_f_ *G*) and the EA were taken from ref 47.

### Thermodynamics in Solution

We attempted to explain the presence of the observed products by using DFT calculations to determine the Gibbs free energies of the various isomers that could be present in the reaction mixture. This is a difficult task due to the presence of more than one phase, the correct definition of standard states, and potentially small energy differences between coexisting species. In Figure 5A, we show the prediction of the thermodynamics of addition of HCl, whereas in Figure 5B, we show the thermodynamics related to the addition of triflic acid. The addition of TfOH is expected to lead to protonation of the **1AgCl** complex. However, this complex could then fall apart, leading to the precipitation of AgCl from of the solution. This is predicted to be energetically favorable by 3 kcal mol^–1^ (admitting that the accuracy of this value relies on the accurate calculation of solvation energies by the COSMO-RS method). The same can be concluded about the addition of HCl, with the important caveat that the precipitation of AgCl from the mixture is predicted to be endergonic by 7.8 kcal mol^–1^ and the most stable state of the system (by ∼3 kcal mol^–1^) is predicted to be one of the two **1AgCl**_**2**_ **H**_**X**_ conformers, whose energy difference is negligible. We may thus reiterate that it is not possible to provide unambiguous arguments for the dominant presence of either of the isomers based on the energy. However, we predict computationally that more polar solvents stabilize **1AgCl**_**2**_ **H**_**Ag**_ relative to **1AgCl**_**2**_ **H**_**Cl**_. In particular, **1AgCl**_**2**_ **H**_**Ag**_ is favored by 0.3 kcal mol^–1^ in DCM, 0.5 kcal mol^–1^ in water, and disfavored by 1.8 kcal mol^–1^ in the gas phase (all values are calculated at the DCM-optimized geometries). Similarly, the addition of an explicit H_2_O molecule stabilizes **1AgCl**_**2**_ **H**_**Ag**_. Moreover, the geometry optimization of **1AgCl**_**2**_ **H**_**Cl**_ ends up in a minimum corresponding to the **1AgCl**_**2**_ **H**_**Ag**_ conformer when a water molecule is placed on the Cl atom closer to the NH^+^ group. Thus, we postulate that polar solvent conditions might be beneficial to the relative stability of the **1AgCl**_**2**_ **H**_**Ag**_ structure. At the same time, we cannot conclusively state which of the isomers is formed under our experimental conditions, but it is likely that both are present in an equilibrium.

## Theoretical Analysis

### Equilibrium Geometries in the Gas Phase and in Solvent

To understand the origin and physicochemical nature of the (formally) M­(I)···H^+^ interactions, we investigated the studied systems computationally. Specifically, we considered (1) **1AgClH** ^**+**^ (the equilibrium 3-D structure depicted in Figure 6B) and its Cu/Au analogues (Figure 6A–C), (2) **1AgCl**_**2**_ **H**_**Ag**_ and **1AgCl**_**2**_ **H**_**Cl**_, systems evidenced in the solvent, calculated using implicit solvent (Figure 6D,E), and (3) the previously studied **2AuClH** ^**+**^ (gas phase, ref 6, Figure 6F).

**Figure 6:**
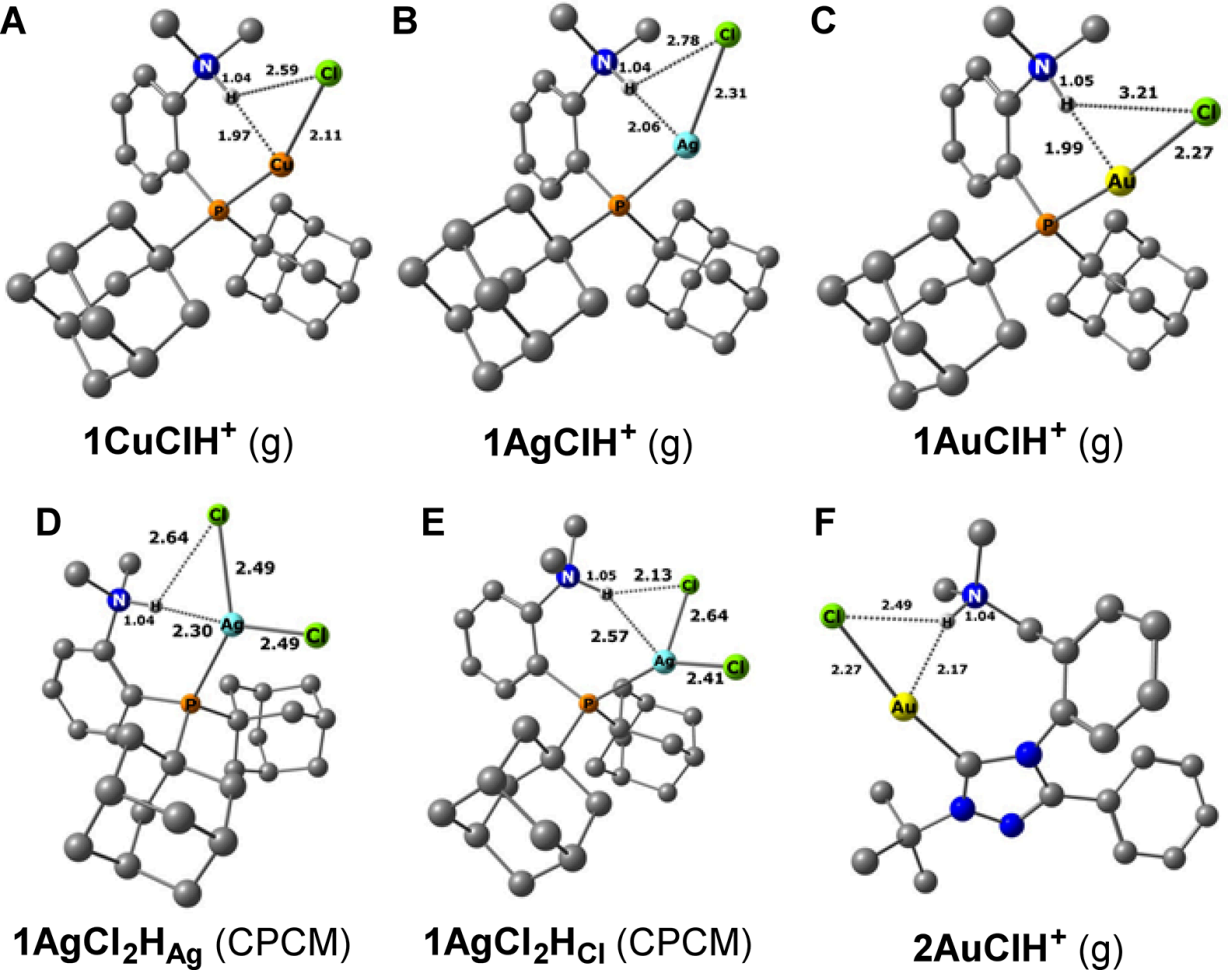
Computed equilibrium structures of **1MClH** ^**+**^ (A–C) in the gas phase, **1AgCl**_**2**_ **H** (D, E) in implicit solvent (CH_2_Cl_2_, CPCM model), and **2AuClH** ^**+**^ (F) in the gas phase. C–H hydrogens were removed for clarity. The PBE0/def2-TZVPP­(ECP_Ag,Au_) method/level was employed. Bond lengths are in Å.

The calculated M···H bond distances for the gas-phase structures of **1MClH** ^**+**^ complexes in Table 2 (1.97 and 2.06 Å) are well within the hydrogen-bonding region. Interestingly, the values exhibit a “zigzag” trend: *R*_M···H_ = 1.97, 2.06, and 1.99 Å for Cu, Ag, and Au, respectively.

The gas-phase *r*_Au···H_ distance in **1AuClH** ^**+**^ is remarkably short. We may remind the reader that the *r*_Au···H_ equilibrium distance in the **2AuClH** ^**+**^ complex was computed to be 2.17 Å (in the gas phase, Figure 6F). We double-checked this peculiarity by employing RI-MP2/def2-TZVP geometry optimization and obtained the same *R*_Au···H_ value of 1.97 Å for **1AuClH** ^**+**^ as in the DFT calculations (see the SI for the equilibrium geometry). In the solvent, the Ag···H bond in **1AgClH** ^**+**^, expectedly, extends from 2.06 to 2.13 Å, but there is still an interaction. Similarly, the *r*_Au···H_ equilibrium distance in **1AuClH** ^**+**^ was predicted to be 2.13 Å when calculated in implicit solvent.^5^

### Analysis of Bonding: Estimating Intramolecular Interaction Energies

Our attempts to provide quantitative arguments complementing the bonding analyses (see below) are based on splitting the studied complexes into the two subsystems (Figure 7). The major structural approximation involves the (in silico) deletion of the phenyl ring that links the dimethylammonium group (interacting with the M^I^ metal ion) with the rest of the **1MClH** ^**+**^ complex. Furthermore, to afford benchmark-quality CCSD­(T)/CBS calculations, we substituted methyls for adamantyls. We expect that this truncation will not influence the relative M­(I)···H^+^ interaction energies (M = Cu, Ag, Au). A similar truncation was done for the previously studied^6^ **2AuClH** ^**+**^, which is also recomputed here for comparison. The same approach was also taken for the interacting dimers of gold­(I) *N-*heterocyclic carbenes (to estimate Au···Au interactions).^48^ To obtain deeper insight into the origin of the M···H interactions, we performed four calculations for each of the model complexes. First, the geometry of the model was fixed at the geometry of the parent complex, and only the hydrogens that capped the open valences (not present in the original system) were optimized at the same level as the parent complexes. This was denoted as **1** ^**modelA**^ **MXH** ^**+**^ (where **1** ^**modelA**^ denotes the small model system originating from ligand **1**, **M** is the metal ion, and **X** is the halogen; in this section, **X** = Cl; cf. Figure 7A). Second, at the final (constrained) geometry of a particular **1** ^**modelA**^ **MXH** ^**+**^ system, the noninteracting proton was removed from the (CH_3_)_2_NH_2_^+^ group to yield neutral **1** ^**modelB**^ **MXH** model system (**1** ^**modelB**^ differs from **1** ^**modelA**^ by the above-described deprotonation, cf. Figure 7B). Neutralizing the interacting species allows for better comparison with the (quantitatively) well-described and standard intermolecular interactions, such as hydrogen and halogen bonds or π–π interactions. Finally, both **1** ^**modelA**^ **MXH** ^**+**^ and **1** ^**modelB**^ **MXH** were fully optimized, the final geometries are denoted as **1** ^**modelA**^ **MXH** ^**+**^_**GM**_ and **1** ^**modelB**^ **MXH**_**GM**_, respectively, where GM stands for global minimum.

**Figure 7:**
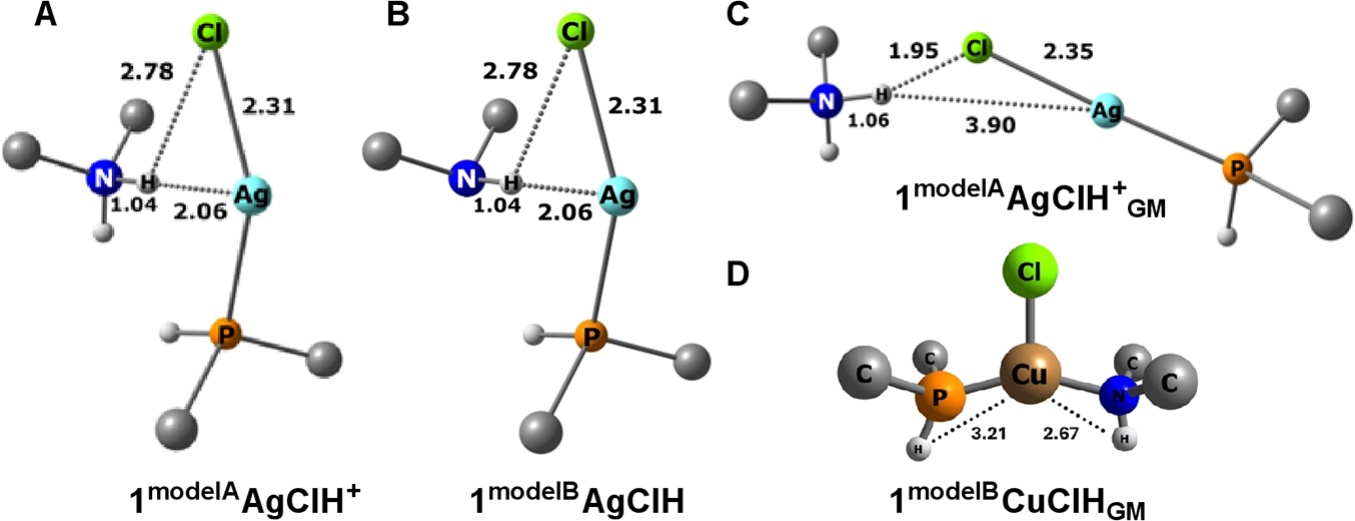
Model systems **1** ^**modelA**^ **AgClH** ^**+**^ (A), **1** ^**modelB**^ **AgClH** (B), **1** ^**modelA**^ **AgClH** ^**+**^_**GM**_ (C), and **1** ^**modelB**^ **CuClH**_**GM**_ (D). Distances are in Å. Methyl hydrogens were removed for clarity.

Structurally, there is nothing interesting in any of the **1** ^**modelA**^ **MClH** ^**+**^ and **1** ^**modelB**^ **MClH** systems, as they were constrained at the geometry of their respective parent complexes (cf., Figures 6 and 7). On the contrary, a major structural rearrangement occurred for all **1** ^**modelA**^ **MClH** ^**+**^_**GM**_ systems (M = Au, Ag, and Cu). Instead of preserving the “relatively weak” M···H interaction, the dimethyl ammonium moiety drifted away and formed the hydrogen bond with the chloride ion (Figure 7C). This is not a surprising finding, at least in the gas phase where the (formally ionic) H^+^···Cl^–^ interaction is expected to be much stronger than the M­(I)**···**H^+^ interaction. This has been observed previously also in the analogous **2** ^**modelA**^ **AuClH** ^**+**^_**GM**_ model of the **2AuClH** ^**+**^ complex.^6^ This structural transition is associated with the energy gain (drop) of ∼20 kcal.mol^–1^, which also (directly) translates to the calculated interaction energies (Table 1). To some extent, surprising structural observations were made for the unconstrained geometry optimizations of the neutral **1** ^**modelB**^ **MClH**_**GM**_ systems. While for Cu and Ag, the equilibrium structures correspond to the trigonal coordination with the phosphine, chloride, and dimethylamine (coordinating via nitrogen) as the ligands (Figure 7D), which could be expected, in the **1** ^**modelB**^ **AuClH**_**GM**_ system, a weak M···H interaction is to some extent preserved. The alternative local minimum corresponding to the P···Au···Cl linear arrangement with only very loosely coordinated (second-sphere) dimethylamine was by 1.2 kcal mol^–1^ higher in energy.

**Table 1:** Computed Interaction Energies for Model Systems (in kcal mol^–1^) at the CCSD­(T)/CBS Level

entry	parent system\model	1^modelA^MClH^+^	1^modelB^MClH	1^modelA^MClH^+^_GM_	1^modelB^MClH_GM_	2^modelA^MClH^+^	full system
1	**1CuClH** ^**+**^	–14.1^a^	–1.6	–32.9	–29.5		
2	**1AgClH** ^+^	–13.7	–1.7	–35.2	–12.3		
3	**1AuClH** ^**+**^	–10.1 (−11.2)	–0.2	–31.2	–7.3		
4	**2AuClH** ^**+**^					–15.1 (−15.8)	
5	(CH_3_)_2_NH_2_^+^···OH_2_						–17.4^b^
6	(CH_3_)_2_NH_2_^+^···C_6_H_6_						–17.4^b^
7	(CH_3_)_2_NH_2_^+^···Xe						–4.3^b^

a X2C two-component relativistic corrected value in parentheses (see Methods).

b Interaction energies (in optimized geometry) of complexes of (CH_3_)_2_NH_2_^+^ with benzene, water, and xenon included for reference.

The computed interaction energies are listed in Table 1, whereas the primary computational data, including various levels of calculations, are available in the Supporting Information (Table S2). Energetically, we consider the **1** ^**modelA**^ **MClH** ^**+**^ systems and their neutral counterparts **1** ^**modelB**^ **MClH** as the most interesting, as they represent the bonding situation in the parent complexes. The estimates of the M···H interaction energy within the parent complexes **1CuClH** ^**+**^ and **1AgClH** ^**+**^ are approximately −14 kcal mol^–1^, whereas it is only approximately −11 kcal mol^–1^ for **1AuClH** ^**+**^. On the contrary, the Au···H interaction amounts to −15.8 kcal mol^–1^ in the **2AuClH** ^**+**^ complex, where we concluded^6^ that the interaction is approaching a strong hydrogen bond. We reiterate that conclusion here by comparing this value (−15.8 kcal mol^–1^) with −17 kcal mol^–1^ computed for the hydrogen-bonded (CH_3_)_2_NH_2_^+^···OH_2_ complex (in its optimal geometry) and with the identical value computed for the (CH_3_)_2_NH_2_^+^···C_6_H_6_ complex. The latter represents the prototypical and presumably strong cation–π interaction. The trends for the **1** ^**modelA**^ **MClH** ^**+**^ model systems are repeated for their neutral **1** ^**modelB**^ **MClH** counterparts (interaction energies of −1.6, −1.7, and −0.2 kcal mol^–1^ for M = Cu, Ag, and Au, respectively). Again, this indicates that there is virtually no Au···H interaction within **1** ^**modelB**^ **MClH**, while Ag/Cu···H interactions are roughly corresponding to the stacking or T-shaped interaction between two benzene rings.^49^ All in all, the computed data clearly confirm weak, but existing, Ag···H and Cu···H interactions in the studied **1MClH** ^**+**^ systems. This agrees with the experimental data presented above. To our surprise, gold­(I) does not seem to be an ideal metal ion to interact with hydrogen in the designed ligand **1** (in contrast to the previously reported **2AuClH** ^**+**^). In our opinion, this can be caused by the shorter equilibrium Au···H distance (which was double checked by two different computational approaches). We may only hypothesize that it originates in the steric requirements of the parent **1AuClH** ^**+**^ system.

### QTAIM Analysis

The results obtained from the QTAIM analysis, focusing specifically on M···H interactions, are listed in Table 2. According to Nakanishi et al.,^50^ typical hydrogen bonds satisfy the following conditions: 0.01 < ϱ < 0.04, 0.04 < Δϱ < 0.12, and −0.004 < *H* < 0.002, where ϱ is the electron density, Δϱ is the Hessian of electron density, and *H* is the kinetic energy density at the corresponding critical point of the M···H or Cl···H bond. The results in Table 2 suggest that both **1CuClH** ^**+**^ and **1AgClH** ^**+**^ feature typical hydrogen bonds in the gas phase, whereas the bonding in **1AuClH** ^**+**^ is on the border between a hydrogen bond and a weak covalent bond. The Ag···H bond in **1AgCl**_**2**_ **H**_**Ag**_ (Figure 6D), which is likely one of the two species that coexist in solution, corresponds to a weak hydrogen bond. The strength of the H···Cl interaction in **1AgCl**_**2**_ **H**_**Cl**_ (Figure 6E) then corresponds to a hydrogen bond; the parameters are listed in the footnote d of Table 2.

**Table 2:** M···H Bond Distances^a^ in Å, QTAIM Parameters of the M···H Bond Critical Points, and NPA Charges of M and H

QTAIM parameters of M···H BCP^b^							NPA charge			
entry	system	*d*(M-H) [Å]	DI	ϱ	Δϱ	*H*	classification	*q*(M)	*q*(H)	χ(M)^c^
1	**1CuClH** ^**+**^(g)	1.97	0.12	0.034	0.072	–0.0037	HB	0.32	0.46	1.90
2	**1AgClH** ^**+**^(g)	2.06	0.13	0.037	0.079	–0.0059	HB	0.43	0.45	1.93
3	**1AuClH** ^**+**^(g)	1.99	0.20	0.054	0.086	–0.0141	weak cov	0.17	0.43	2.54
4	**1AgCl**_**2**_ **H**_**Ag**_ (CPCM)	2.30	0.08	0.024	0.049	–0.0008	HB	0.36	0.48	
5	**1AgCl**_**2**_ **H**_**Cl**_ (CPCM)^d^	2.57	-	-	-	-	-	0.35	0.51	
6	**2AuClH** ^**+**^(g)	2.17	0.13	0.036	0.076	–0.0041	HB	0.26	0.46	
7	^**M**^ **1CuClH** ^**+**^(g)	1.97	0.12	0.034	0.073	–0.0034	HB	0.34	0.43	1.90
8	^**M**^ **1AgClH** ^**+**^(g)	2.06	0.13	0.037	0.079	–0.0057	HB	0.42	0.42	1.93
9	^**M**^ **1AuClH** ^**+**^(g)	1.99	0.20	0.054	0.086	–0.0140	weak cov	0.23	0.40	2.54
10	**2AuClH** ^**+**^(g)	2.17	0.13	0.036	0.076	–0.0040	HB	0.30	0.45	

a Molecular structures and QTAIM were computed at the PBE0-D3/def2TZVPP/ECP­(M) level in vacuo at 0 K, “gas-phase”, (g), or with implicit solvent model (CPCM), see Methods.

b Analysis was carried out at the PBE0/def2TZVP level, “gas phase”, or CPCM. BCP is the bond critical point, ϱ is the electron density, Δϱ is the Laplacian of electron density, and *H* is the electron energy density for given BCP. According to ref 50, typical hydrogen bonds satisfy 0.01 < ϱ < 0.04, 0.04 < Δϱ < 0.12, −0.004 < *H* < 0.002. Lower values of ϱ correspond to a weak van der Waals (vdW) bond and higher ϱ values to charge transfer or covalent bonds. DI is the delocalization index that quantifies the electron density shared by two atoms.

c Pauling electronegativity.^51^

d No critical point is observed for Ag···H bonding. The corresponding values for NH···Cl critical point are DI = 0.13, ϱ = 0.033, Δϱ = 0.075, and *H* – 0.0018, which classifies as a hydrogen bond.

Another parameter provided by QTAIM is the delocalization index (DI). It quantifies the number of electron pairs shared between two atoms. DI is arguably the simplest parameter that measures the mutual interaction between two atoms, regardless of how the interaction is formally classified. The computed DI (Table 2) confirms the existence of M···H interactions in the **1MClH** ^**+**^(g) complexes (items 1–3) and weaker interactions in the solvated **1AgCl**_**2**_ **H**_**Ag**_ system. The order of computed DIs in the former systems reflects the electronegativity of the metal (see the last column in Table 2), with the most electronegative gold having the strongest interaction. The gold also has the smallest positive charge of all three metal ions (NPA charge of 0.17), as expected; the second least positive NPA charge belongs to copper and not silver. The high electronegativity of coinage metals (particularly gold) together with their propensity for low coordination number and polarizability (softness) of the metal are, in fact, the factors that enable metal–hydrogen bonds. Owing to the electronegativity, and hence low partial charge on metal, the electrostatic repulsion with hydrogen is low, while the dispersion and orbital interaction is high. Furthermore, the preference for the low coordination number implies that there is an open space in the coordination sphere to form a hydrogen bond.

Interestingly, the computed QTAIM parameters for **1AuClH** ^**+**^(g) indicate a “stronger” M···H bonding interaction compared to Cu and Ag. At first glance, this may appear to be inconsistent with the computed interaction energies for the model systems (Table 1). However, this is not a true contradiction, but rather a reflection of the fact that the two approaches quantify different aspects of the system. Interaction energies account for the total stabilization arising from all interactions between the two subsystems, while QTAIM focuses on the topology of the electron density and identifies critical points between specific pairs of atoms. Consequently, interaction energies can serve as a useful, but coarse measure of bonding, whereas QTAIM provides a localized description of electron density features. It is also important to note that QTAIM parameters, while insightful for bonding analysis, are not direct quantum mechanical observables.

As an alternative, we also carried out EDA and NOECV analyses (see Table S3, Figure S15, and the accompanying discussion in the Supporting Information). The general trends observed in QTAIM are reproduced by EDA and NOECV. At the same time, EDA suggests that the steric interaction term is by ∼7 kcal mol^–1^ more positive in the case of **1AuClH** ^**+**^ (g), thus destabilizing the overall interaction compared with its Ag and Cu counterparts. Albeit qualitatively, this appeases the apparent discrepancy between the less favorable interaction energies computed for the Au models (with respect to the Cu and Ag models) and QTAIM analysis, which indicated stronger M···H interactions for Au (vide supra).

## Discussion

We used multiple experimental and computational methods to address the question whether silver­(I) and copper­(I) may act as hydrogen-bond acceptors in the gas phase or in solution. Predicting computationally that ligand **1** can be a suitable candidate for observing these unprecedented interactions, we first characterized the ensuing complexes in the gas phase. Infrared spectra in the gas phase (IRPD spectra) of **1AgClH** ^**+**^ and **1CuIH** ^**+**^ look overall quite similar, which reflects the (expected) similar structures of the complexes. We expect that the substitution of Cl for I in the complex, which was done solely due to better availability of CuI, to have a little effect on the metal–hydrogen bond by analogy with a series of Au­(I) complexes that we studied previously.^14^ Based on the comparison of the spectrum of **1AgClH** ^**+**^ with GVPT2 calculations, we assigned the 2968 cm^–1^ band in its spectrum as the N–H stretching band (ν­(NH) band) and the two bands around 2700 cm^–1^ as overtones of the N–H bending vibrational modes (δ­(NH) modes). Under harmonic approximation, the ν­(NH) band in the copper complex should be ∼100 cm^–1^ lower than that in the silver complex. The GVPT2 calculations, however, predict that the band frequency should shift lower to 2694 cm^–1^ and thus overlap with the δ­(NH) overtone region. This is quite interesting, because the difference in bond strength between the two metals is expected to be rather small, as indicated by entries 1 and 2 in Table 2 and entries 1 and 2 in Table 1. Our only conclusion is that the N–H bond in CuI is much more anharmonic, for unknown reasons.

If we compare the spectra of **1AgClH** ^**+**^ to our previously measured **2AuClH** ^**+**^ complex, which features the ν­(NH) band around 2700 cm^–1^, the Ag···H interaction in **1AgClH** ^**+**^ is likely weaker than that of Au···H in **2AuClH** ^**+**^. This agrees with calculated interaction energies for model systems (entries 2 and 4 in Table 1). However, we cannot make any definitive conclusions for **1CuIH** ^**+**^ interaction strength, due to limitations of GVPT2 calculation, which had encountered convergence problems and also had problem reproducing observed peak intensities, with the intensity of some C–H bands predicted to be very high, despite being experimentally the same as in the silver complex (Figure 2E). However, we believe that we can still conclude that the observed IRPD spectra are generally consistent with the structures featuring M···H hydrogen bonding interactions.

Since the utility and relevance of M···H interactions would be much greater if they existed in the condensed phase, we attempted to verify if such interactions could also be present in solution. To this aim, we focused our efforts on the silver complex because silver has a greater tendency to form linear coordination that allows the formation of H bonds and it has a lower tendency to oxidize than Cu­(I) complexes. In addition, it can be conveniently studied by employing ^109^Ag NMR spectroscopy because of its nuclear spin of 1/2. We performed a search of the Cambridge Structural Database^52^ for possible Ag···H­(N) contacts and identified 8 structures, where such contacts may exist (Table S4), but the true nature of these, including the exact position of hydrogens, is uncertain and would require more study.

Coordination of the metal center with additional ligands reduces the likelihood that H bonding will be energetically favorable. Upon the addition of HCl, we observed NMR peaks that confirm the protonation of the dimethylamino unit and still show the presence of a Ag–P bond in the complex. Calculated chemical shifts of Ag and P were best consistent with a structure **1AgCl**_**2**_ **H**. We also observed that the NH proton moved to the ∼10 ppm range, which is similar to 10.9 ppm observed for the Au···HN proton by Rigoulet et al.^5^ DFT calculations identified two isoenergetic conformers of **1AgCl**_**2**_ **H**, where the NH forms a hydrogen bond with either Ag or Cl atom. Solution NMR data that we obtained cannot distinguish between these conformers, and the most likely conclusion is that both are likely present in solution. If that is correct, it would present a curious example where the Ag···H­(N^+^) bond is competitive to the Cl···H­(N^+^) bond in solution. A limitation of the current finding is the low stability of the complex, which decomposes above −50 °C. In this regard, it is also interesting to note that the interaction energies of the (CH_3_)_2_NH_2_^+^ unit with the [((CH_3_)_2_(H)­P)­AgCl] unit in the model **1AgClH** ^**+**^ complex (−13.7 kcal mol^–1^) are only 3 kcal mol^–1^ weaker than in the corresponding complex with water (featuring prototypical and strong hydrogen bond) and 9 kcal mol^–1^ stronger than the Xe···(CH_3_)_2_NH_2_^+^ interaction. Surprisingly, it is 3.6 kcal mol^–1^ stronger than the same interaction within **1AuClH** ^**+**^ (admitting that this lower value might originate in the steric repulsions, as also indicated by the EDA analysis, Table S3). Therefore, we believe that the low stability of the **1AgCl**_**2**_ **H** complex might be due to the large lattice energy of solid AgCl, which we observed to precipitate out of solution in our experiments, rather than due to the low strength of the Ag···H bond.

## Conclusions

In this work, we presented spectroscopic and computational evidence of hydrogen bonds to silver in the gas phase and in solution. We showed that upon coordination of the AgCl unit to the rigid phosphine ligand **1** and subsequent protonation in the gas phase, a structure with Ag···H contact was formed, as evidenced from the gas-phase IR spectra of the corresponding complex **1AgClH** ^**+**^. Various bonding analyses showed that the Ag···H interaction is best described as a weak, but classical, hydrogen bond, rather than other types of interactions (like those observed for M···H­(C) contacts^15^). We also attempted to form the **1AgClH** ^**+**^ complex in solution but upon addition of TfOH to **1AgCl**, we only observed the formation of the protonated **1H** ^**+**^ ligand. However, after the addition of HCl, we observed a silver complex that was eventually assigned to be **1AgCl**_**2**_ **H** ^**+**^. This complex has two isoenergetic forms, one of which features a Ag···H­(N^+^) bond. Although unstable (the complex decomposed above −50 °C), this represents a rare example of such interaction in the condensed phase, acknowledging that a number of crystal complexes with short Ag···H contacts can be found in the crystallographic databases. We also present the experimental gas-phase data for the **1CuIH** ^**+**^ complex that might suggest a structure similar to silver, but the nonperfect agreement between the observed IR spectra and our calculations does not allow us to claim the presence of Cu···H interaction with certainty. All in all, we consider the interactions of coinage metals with hydrogen as an important phenomenon that should be considered in various areas of chemical research, alongside their better characterized aurophilic counterparts.

## Supplementary Material







## Notes

XYZ coordinates of reported structures, calculated at the PBE0/def2TZVPP (COSMO, ε = 9) level unless indicated otherwise; the naming in some cases is according to Figure S14 (XYZ)
Calculated anharmonic spectra used to create Figure 2 (ZIP)
ESI-MS spectrum (Figure S1); collision-included dissociation spectrum (Figure S2); ^1^H NMR spectrum (Figures S3 and S12); aromatic region of ^1^H NMR spectrum (Figure S4); ROESY spectrum (Figure S5); ^13^C APT NMR spectra (Figure S6); ^31^P NMR spectrum (Figures S7, S8, and S13); aromatic and PH region of ^1^H NMR spectrum (Figure S9); aromatic and NH region of ^1^H NMR spectrum (Figure S10); aromatic and NH region of ^1^H,^15^N-HSQC spectrum (Figure S11); equilibrium geometries (Table S1); calculated NMR parameters (Figure S14); energies of the model systems (Table S2); EDA decomposition and NOCV for model systems (Table S3); M···H interacting NOCV (Figure S15); and close Ag···H-N contacts in the CSD (Table S4) (PDF)

## References

[1] Schmidbaur H., Raubenheimer H. G., Dobrzańska L. (2014). The gold–hydrogen bond, Au–H,
and the hydrogen bond to gold, Au···H–X. Chem. Soc. Rev..

[2] Vícha J., Foroutan-Nejad C., Straka M. (2019). 1H NMR is not a proof of hydrogen
bonds in transition metal complexes. Nat. Commun..

[3] Berger R. J. F., Schoiber J., Monkowius U. (2017). A Relativity
Enhanced, Medium-Strong
Au­(I)···H–N Hydrogen Bond in a Protonated Phenylpyridine-Gold­(I)
Thiolate. Inorg. Chem..

[4] Groenewald F., Raubenheimer H. G., Dillen J., Esterhuysen C. (2017). Gold setting
the “gold standard” among transition metals as a hydrogen
bond acceptor – a theoretical investigation. Dalton Trans..

[5] Rigoulet M., Massou S., Sosa Carrizo E. D., Mallet-Ladeira S., Amgoune A., Miqueu K., Bourissou D. (2019). Evidence for
genuine hydrogen bonding in gold­(I) complexes. Proc. Natl. Acad. Sci. U.S.A..

[6] Straka M., Andris E., Vícha J., Růžička A., Roithová J., Rulíšek L. (2019). Spectroscopic and Computational
Evidence of Intramolecular Au^I^···H^+^–N Hydrogen Bonding. Angew. Chem., Int.
Ed..

[7] Darmandeh H., Löffler J., Tzouras N. V., Dereli B., Scherpf T., Feichtner K., Vanden Broeck S., Van Hecke K., Saab M., Cazin C. S. J., Cavallo L., Nolan S. P., Gessner V. H. (2021). Au···H–C
Hydrogen Bonds as Design
Principle in Gold­(I) Catalysis. Angew. Chem.,
Int. Ed..

[8] Sorroche A., Moreno S., Elena Olmos M., Monge M., López-de-Luzuriaga J. M. (2023). Deciphering
the Primary Role of Au···H–X Hydrogen Bonding
in Gold Catalysis. Angew. Chem., Int. Ed..

[9] Sahu K., Mondal S., Mobin S. M., Kar S. (2021). Photocatalytic
C–H
Thiocyanation of Corroles: Development of Near-Infrared (NIR)-Emissive
Dyes. J. Org. Chem..

[10] Feng X., Yang J., Miao J., Zhong C., Yin X., Li N., Wu C., Zhang Q., Chen Y., Li K., Yang C. (2022). Au···H–C Interactions Support a Robust Thermally
Activated Delayed Fluorescence (TADF) Gold­(I) Complex for OLEDs with
Little Efficiency Roll-Off and Good Stability. Angew. Chem., Int. Ed..

[11] Ma X., Li J., Luo P., Hu J., Han Z., Dong X., Xie G., Zang S. (2023). Carbene-stabilized enantiopure heterometallic clusters
featuring EQE of 20.8% in circularly-polarized OLED. Nat. Commun..

[12] Li Y., Chen Y., Liu Z. (2022). OH^–^···Au
Hydrogen Bond and Its Effect on the Oxygen Reduction Reaction on Au(100)
in Alkaline Media. J. Phys. Chem. Lett..

[13] Vávrová A., Čapková T., Kuřitka I., Vícha J., Münster L. (2022). One-step synthesis
of gold nanoparticles
for catalysis and SERS applications using selectively dicarboxylated
cellulose and hyaluronate. Int. J. Biol. Macromol..

[14] Andris E., Straka M., Vrána J., Růžička A., Roithová J., Rulíšek L. (2023). Can Copper­(I) and Silver­(I)
be Hydrogen Bond Acceptors?. Chem. Eur J..

[15] Raubenheimer H. G., Dobrzańska L. (2020). Interaction between Cu and Ag free
ions and central
metals in complexes with XHn units (X = B, Si, N, O, C, Al, Zn, Mg;
n = 1, 2). Coord. Chem. Rev..

[16] dos
Passos Gomes G., Xu G., Zhu X., Chamoreau L., Zhang Y., Bistri-Aslanoff O., Roland S., Alabugin I. V., Sollogoub M. (2021). Mapping C–H···M Interactions
in Confined Spaces: (α-ICyD^Me^)­Au, Ag, Cu Complexes
Reveal “Contra-electrostatic H Bonds” Masquerading as
Anagostic Interactions. Chem. Eur J..

[17] Mannarsamy M., Prabusankar G. (2021). Rare proximity enforced copper hydrogen
interactions
in copper­(i)-chalcogenones. New J. Chem..

[18] Sahu K., Dutta J., Nayak S., Nayak P., Biswal H. S., Kar S. (2022). Investigation of the Nature of Intermolecular Interactions in Tetra­(thiocyanato)­corrolato-Ag­(III)
Complexes: Agostic or Hydrogen Bonded?. Inorg.
Chem..

[19] Sorroche A., Reboiro F., Monge M., López-de-Luzuriaga J. M. (2024). Recent
Trends in Group 11 Hydrogen Bonding. ChemPlusChem.

[20] Roithová J., Gray A., Andris E., Jašík J., Gerlich D. (2016). Helium Tagging Infrared Photodissociation Spectroscopy
of Reactive Ions. Acc. Chem. Res..

[21] Jašík J., Žabka J., Roithová J., Gerlich D. (2013). Infrared spectroscopy
of trapped molecular dications below 4K. Int.
J. Mass Spectrom..

[22] Frisch, M. J. ; Trucks, G. W. ; Schlegel, H. B. ; Scuseria, G. E. ; Robb, M. A. ; Cheeseman, J. R. ; Scalmani, G. ; Barone, V. ; Petersson, G. A. ; Nakatsuji, H. ; Li, X. ; Caricato, M. ; Marenich, A. V. ; Bloino, J. ; Janesko, B. G. ; Gomperts, R. ; Mennucci, B. ; Hratchian, H. P. ; Ortiz, J. V. ; Izmaylov, A. F. ; Sonnenberg, J. L. ; Williams-Young, D. ; Ding, F. ; Lipparini, F. ; Egidi, F. ; Goings, J. ; Peng, B. ; Petrone, A. ; Henderson, T. ; Ranasinghe, D. ; Zakrzewski, V. G. ; Gao, J. ; Rega, N. ; Zheng, G. ; Liang, W. ; Hada, M. ; Ehara, M. ; Toyota, K. ; Fukuda, R. ; Hasegawa, J. ; Ishida, M. ; Nakajima, T. ; Honda, Y. ; Kitao, O. ; Nakai, H. ; Vreven, T. ; Throssell, K. ; Montgomery, J. A., Jr. ; Peralta, J. E. ; Ogliaro, F. ; Bearpark, M. J. ; Heyd, J. J. ; Brothers, E. N. ; Kudin, K. N. ; Staroverov, V. N. ; Keith, T. A. ; Kobayashi, R. ; Normand, J. ; Raghavachari, K. ; Rendell, A. P. ; Burant, J. C. ; Iyengar, S. S. ; Tomasi, J. ; Cossi, M. ; Millam, J. M. ; Klene, M. ; Adamo, C. ; Cammi, R. ; Ochterski, J. W. ; Martin, R. L. ; Morokuma, K. ; Farkas, O. ; Foresman, J. B. ; Fox, D. J. Gaussian 16, revision C.01; Gaussian, Inc.: Wallingford CT, 2016.

[23] Stephens P. J., Devlin F. J., Chabalowski C. F., Frisch M. J. (1994). Ab Initio Calculation
of Vibrational Absorption and Circular Dichroism Spectra Using Density
Functional Force Fields. J. Phys. Chem..

[24] Becke A. D. (1993). Density-functional
thermochemistry. III. The role of exact exchange. J. Chem. Phys..

[25] Lee C., Yang W., Parr R. G. (1988). Development
of the Colle-Salvetti
correlation-energy formula into a functional of the electron density. Phys. Rev. B.

[26] Vosko S. H., Wilk L., Nusair M. (1980). Accurate spin-dependent
electron
liquid correlation energies for local spin density calculations: a
critical analysis. Can. J. Phys..

[27] Grimme S., Antony J., Ehrlich S., Krieg H. (2010). A consistent and accurate *ab initio* parametrization of density functional dispersion
correction (DFT-D) for the 94 elements H-Pu. J. Chem. Phys..

[28] Grimme S., Ehrlich S., Goerigk L. (2011). Effect of
the damping function in
dispersion corrected density functional theory. J. Comput. Chem..

[29] ADF 2024.1, SCM, Theoretical Chemistry; Vrije Universiteit, Amsterdam, The Netherlands, http://www.scm.com.

[30] Vícha J., Komorovsky S., Repisky M., Marek R., Straka M. (2018). Relativistic
Spin–Orbit Heavy Atom on the Light Atom NMR Chemical Shifts:
General Trends Across the Periodic Table Explained. J. Chem. Theory Comput..

[100] Adamo C., Barone V. (1999). Toward reliable density functional
methods without adjustable parameters: The PBE0 model. J. Chem. Phys..

[31] Vícha J., Novotný J., Komorovsky S., Straka M., Kaupp M., Marek R. (2020). Relativistic Heavy-Neighbor-Atom Effects on NMR Shifts: Concepts
and Trends Across the Periodic Table. Chem.
Rev..

[32] Bader R. F. W. (1991). A quantum
theory of molecular structure and its applications. Chem. Rev..

[33] Keith, T. A. AIMALL. version 19.10.12; TK Gristmill Software: Overland Park KS: USA, 2019.

[34] Michalak A., Mitoraj M., Ziegler T. (2008). Bond Orbitals from
Chemical Valence
Theory. J. Phys. Chem. A.

[35] Glendening E. D., Landis C. R., Weinhold F. (2012). Natural bond orbital methods. Wiley Interdiscip. Rev.: Comput. Mol. Sci..

[36] TURBOMOLE V7.8 2023, a development of University of Karlsruhe and Forschungszentrum Karlsruhe GmbH, 1989–2007, TURBOMOLE GmbH, since, 2007; available from https://www.turbomole.org.

[37] Balasubramani S. G., Chen G. P., Coriani S., Diedenhofen M., Frank M. S., Franzke Y. J., Furche F., Grotjahn R., Harding M. E., Hättig C., Hellweg A., Helmich-Paris B., Holzer C., Huniar U., Kaupp M., Marefat
Khah A., Karbalaei Khani S., Müller T., Mack F., Nguyen B. D., Parker S. M., Perlt E., Rappoport D., Reiter K., Roy S., Rückert M., Schmitz G., Sierka M., Tapavicza E., Tew D. P., van Wüllen C., Voora V. K., Weigend F., Wodyński A., Yu J. M. (2020). TURBOMOLE: Modular program suite
for *ab initio* quantum-chemical and condensed-matter
simulations. J. Chem. Phys..

[38] Dunning T.
H. (1989). Gaussian
basis sets for use in correlated molecular calculations. I. The atoms
boron through neon and hydrogen. J. Chem. Phys..

[39] Kendall R. A., Dunning T. H., Harrison R. J. (1992). Electron affinities of the first-row
atoms revisited. Systematic basis sets and wave functions. J. Chem. Phys..

[40] Woon D. E., Dunning T. H. (1993). Gaussian basis sets
for use in correlated molecular
calculations. III. The atoms aluminum through argon. J. Chem. Phys..

[41] Peterson K.
A., Puzzarini C. (2005). Systematically
convergent basis sets for transition
metals. II. Pseudopotential-based correlation consistent basis sets
for the group 11 (Cu, Ag, Au) and 12 (Zn, Cd, Hg) elements. Theor. Chem. Acc..

[42] Andrae D., Haussermann U., Dolg M., Stoll H., Preuß H. (1990). Energy-adjusted *ab-initio* pseudopotentials for the second and third row
transition elements. Theor. Chim. Acta.

[43] Peng D., Middendorf N., Weigend F., Reiher M. (2013). An efficient implementation
of two-component relativistic exact-decoupling methods for large molecules. J. Chem. Phys..

[44] Pollak P., Weigend F. (2017). Segmented Contracted
Error-Consistent Basis Sets of
Double- and Triple-ζ Valence Quality for One- and Two-Component
Relativistic All-Electron Calculations. J. Chem.
Theory Comput..

[45] Procházková E., Šimon P., Straka M., Filo J., Májek M., Cigáň M., Baszczyňski O. (2021). Phosphate linkers with traceable
cyclic intermediates for self-immolation detection and monitoring. Chem. Commun..

[101] Neese F. (2025). Software Update: The ORCA Program
System-Version 6.0. WIREs Comput. Mol. Sci..

[102] Klamt A., Jonas V., Burger T., Lohrenz J. C. W. (1998). Refinement
and Parametrization of COSMO-RS. J. Phys. Chem.
A.

[103] Becke A.
D. (1988). Density-Functional Exchange-Energy
Approximation with
Correct Asymptotic Behavior. Phys. Rev. A.

[104] Perdew J. P. (1986). Density-Functional Approximation
for the Correlation
Energy of the Inhomogeneous Electron Gas. Phys.
Rev. B.

[105] Klamt A., Diedenhofen M. (2018). A refined cavity construction algorithm
for the conductor-like screening model. J. Comput.
Chem..

[46] Loock H., Beaty L., Simard B. (1999). Reassessment
of the first ionization
potentials of copper, silver, and gold. Phys.
Rev. A.

[47] CRC Handbook of Chemistry and Physics; 88th ed., Lide, D. R. , Ed.; CRC Press: Boca Raton, 2007–2008; pp. 5–1,10–156

[48] Andris E., Andrikopoulos P. C., Schulz J., Turek J., Růžička A., Roithová J., Rulíšek L. (2018). Aurophilic Interactions
in [(L)­AuCl]···[(L′)­AuCl] Dimers: Calibration
by Experiment and Theory. J. Am. Chem. Soc..

[49] Pitoňák M., Neogrády P., Řezáč J., Jurečka P., Urban M., Hobza P. (2008). Benzene Dimer: High-Level Wave Function
and Density Functional Theory Calculations. J. Chem. Theory Comput..

[50] Nakanishi W., Hayashi S., Narahara K. (2008). Atoms-in-Molecules Dual Parameter
Analysis of Weak to Strong Interactions: Behaviors of Electronic Energy
Densities versus Laplacian of Electron Densities at Bond Critical
Points. J. Phys. Chem. A.

[51] Allred A. (1961). Electronegativity
values from thermochemical data. J. Inorg. Nucl.
Chem..

[52] Groom C. R., Bruno I. J., Lightfoot M. P., Ward S. C. (2016). The Cambridge Structural
Database. Acta Crystallogr., B Struct. Sci.
Cryst. Eng. Mater..

