# Supplementary material for: Can Silver(I) Act as a Hydrogen-Bond Acceptor? Spectroscopic and Computational Exploration of the Ag···H+ Bonds in the Gas Phase and in Solvent
Source: Inorg Chem. 2025 Dec 8;64(50):24411–22. doi: 10.1021/acs.inorgchem.5c03415 (PMC12728918; doi:10.1021/acs.inorgchem.5c03415)
Supplement: Supplementary file 3 [file ic5c03415_si_003.pdf]

Supporting Information

Can Silver(I) Act as a Hydrogen-Bond Acceptor? Spectroscopic and Computational Exploration of the Ag...H$^+$ Bonds in the Gas-Phase and in Solvent

Erik Andris,[*,†] Michal Straka,[†] Martin Dračínský,[†] Qin Yang,[†] Jana Roithová,[‡] Lubomír Rulíšek[†]

[†] Institute of Organic Chemistry and Biochemistry of the Czech Academy of Sciences, Flemingovo náměstí 2, 16610 Praha 6, Czech Republic. Email: erik.andris@uochb.cas.cz

[‡] Institute for Molecules and Materials, Radboud University, Heyendaalseweg 135, 6525 AJ Nijmegen, The Netherlands

Mass Spectra

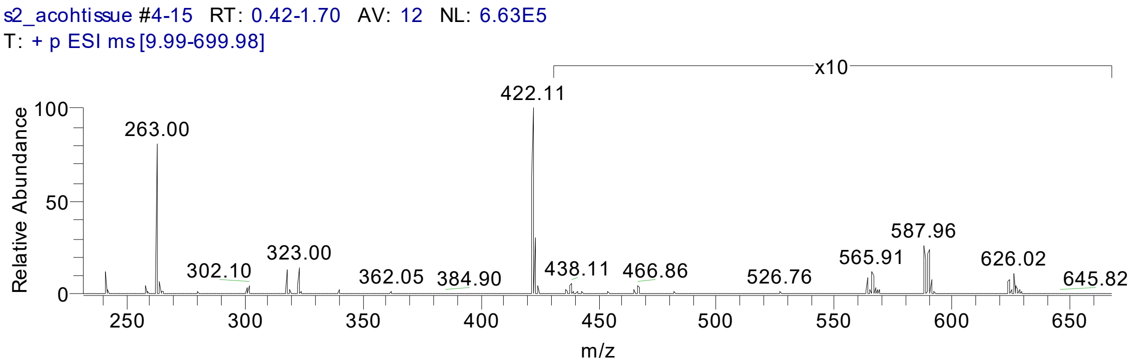

Figure S1. ESI-MS spectrum of **1AgCl** sprayed with AcOH in the ion source. Collision induded dissociation spectrum of **1AgClH**$^+$ ion (m/z 564) is shown in Figure S2.

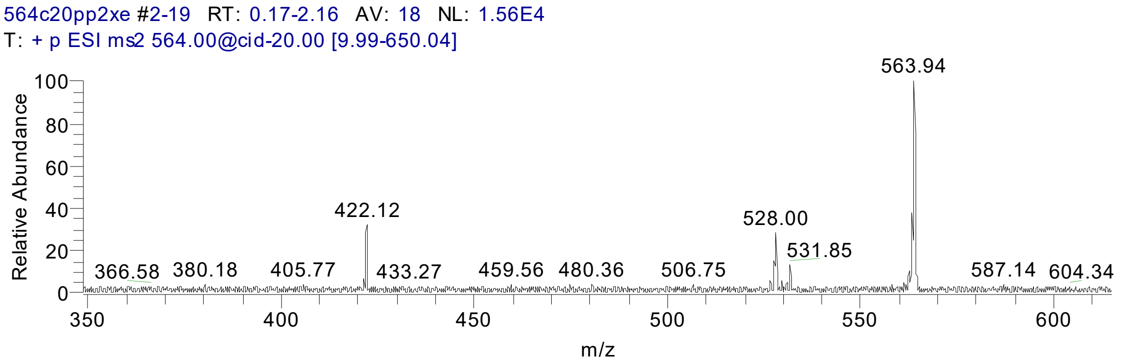

Figure S2 . CID of m/z 564. The peak at m/z 532 likely corresponds to loss of MeOH from isobaric impurity of **1AgOMe**$^+$ with ^{109}Ag atom and one ^{13}C atom, whereas the peak at m/z 528 corresponds to the loss of H^{35}Cl and the peak at m/z 422 corresponds to the loss of ^{107}Ag^{35}Cl.

NMR spectra

List of NMR shifts and *J*-coupling constants for 1AgCl.

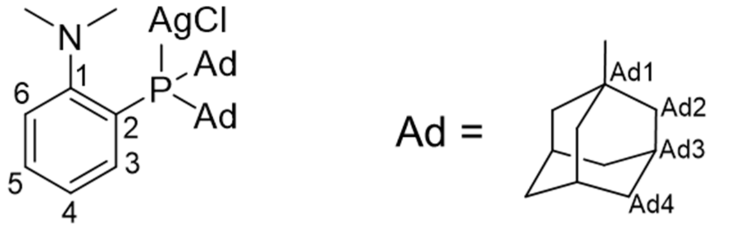

^{1}H NMR (CD_2Cl_2, 500.3 MHz, 25 °C): δ = 7.80 (ddd, 1H, J(H3,H4) = 7.9, J(H3,P) = 4.3, J(H3,H5) = 1.6, H3), 7.58 (ddd, 1H, J(H6,H5) = 8.2, J(H6,P) = 4.7, J(H6,H4) = 1.6, H6), 7.54 (m, 1H, H5), 7.29 (ddd, 1H, J(H4,H3) = 8.2, J(H4,H5) = 7.2, J(H4,H6) = 1.6, H4), 2.73 (s, 6H, CH_3), 2.17 (m, 6H, Ad2), 1.94–2.01 (m, 12H, Ad2, Ad3), 1.67–1.74 (m, 12H, Ad4).
^{13}C NMR (CD_2Cl_2, 125.8 MHz, 25 °C): δ = 160.9 (dd, J(C1,P) = 12.8, J(C1,Ag) = 2.3, C1), 136.1 (dd, J(C3,Ag) = 5.9, J(C3,P) = 1.7, C3), 132.3 (s, C5), 126.5 (dd, J(C2,P) = 25.2, J(C2,Ag) = 2.6, C2), 125.3 (dd, J(C4,P) = 3.3, J(C4,Ag) = 1.4, C4), 125.2 (d, J(C6,P) = 3.3, C6), 49.4 (s, NCH_3), 42.3 (dd, J(Ad2,P) = 7.3, J(Ad2,Ag) = 1.9, Ad2), 39.2 (dd, J(Ad1,P) = 4.6, J(Ad1,Ag) = 4.3, Ad1), 36.8 (d, J(Ad4,P) = 1.4, Ad4), 29.2 (d, J(Ad3,P) = 9.8, Ad3).

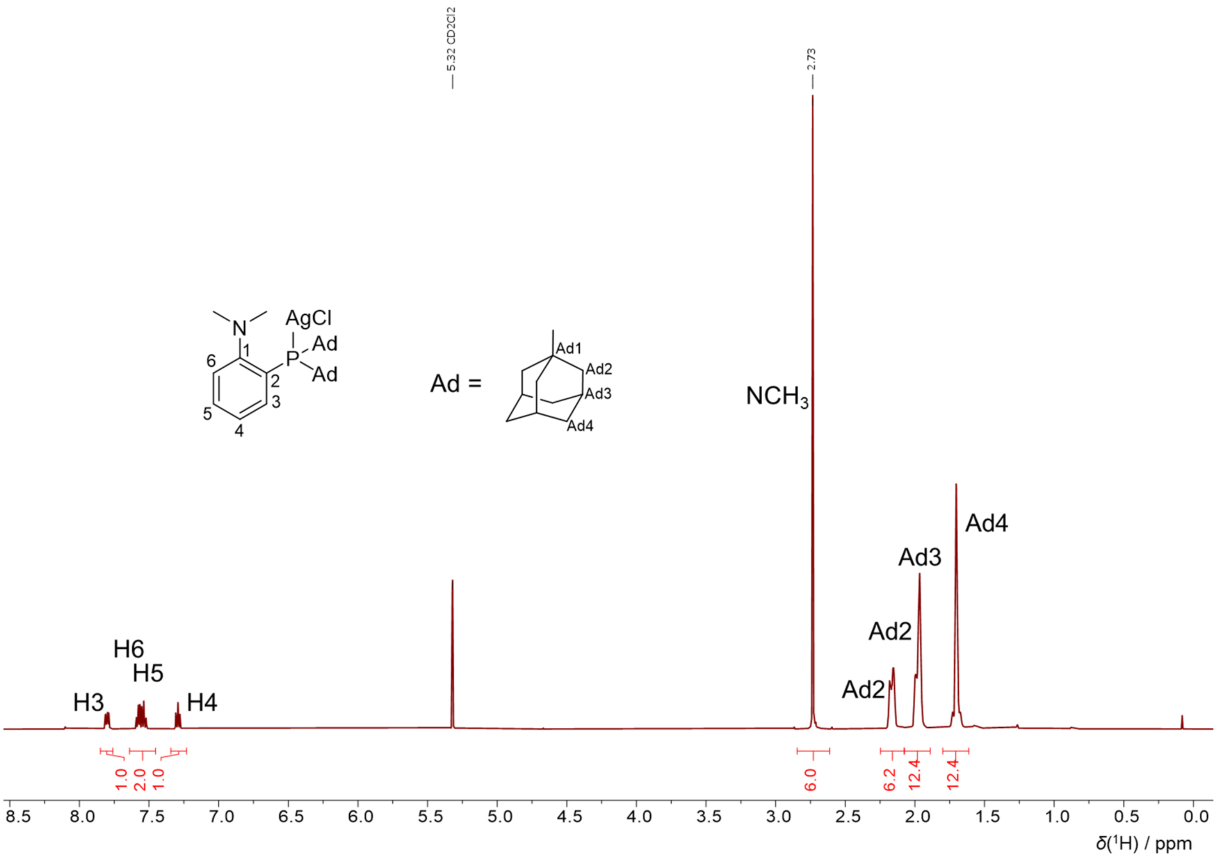

Figure S3. ^{1}H NMR spectrum of **1AgCl** in CD_2Cl_2 at 25 °C.

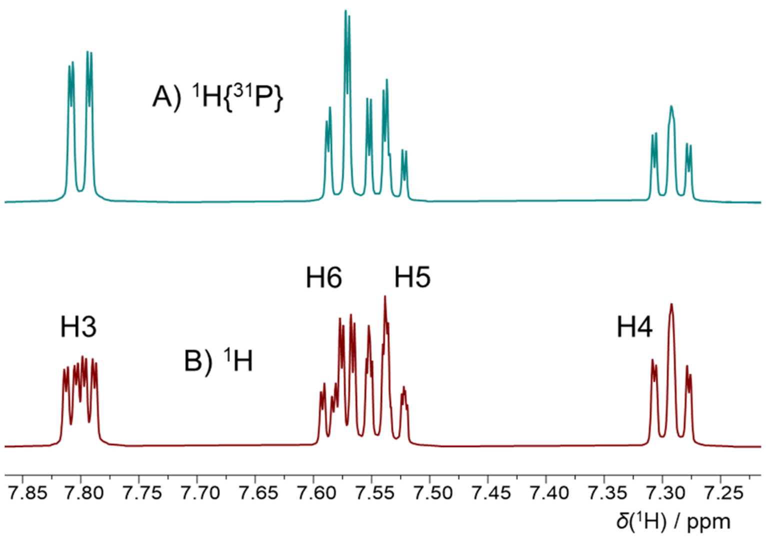

Figure S4. The aromatic region of ^{1}H NMR spectrum of **1AgCl** in CD_2Cl_2 at 25 °C with A) ^{31}P decoupling and B) without decoupling. This experiment reveals long-range ^{1}H,^{31}P couplings ^{3}J(H3,P) = 4.3 Hz, ^{4}J(H6,P) = 4.7 Hz, ^{5}J(H3,P) = 0.8 Hz.

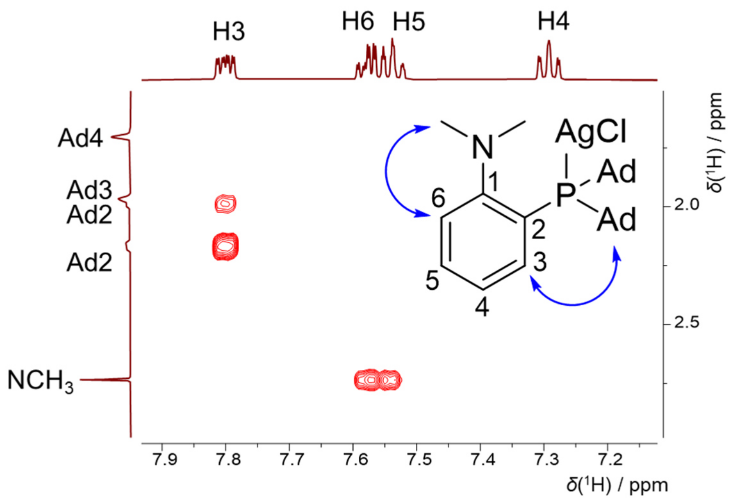

Figure S5. ROESY spectrum of **1AgCl** in CD_2Cl_2 at 25 °C showing the indicated spatial proximities of hydrogen atoms.

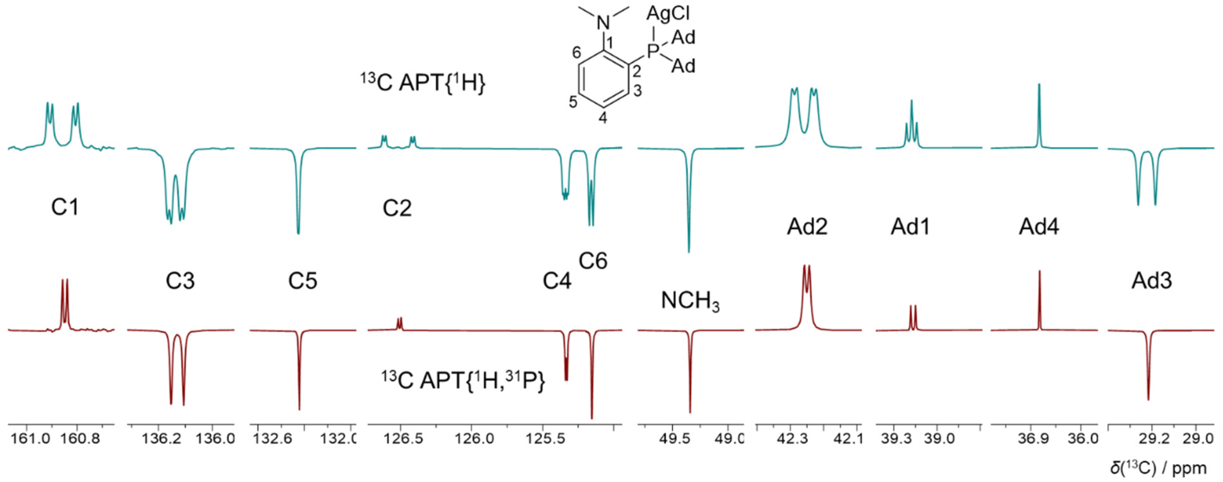

Figure S6. ^{13}C APT NMR spectra of **1AgCl** in CD_2Cl_2 at 25 °C with ^{1}H decoupling only and with ^{1}H and ^{31}P decoupling. These experiments reveal short- and long-range ^{13}C,^{31}P and ^{13}C,$^{107/109}$Ag couplings ^{2}J(C1,P) = 12.8 Hz, ^{2}J(C3,P) = 1.7 Hz, ^{1}J(C2,P) = 25.2 Hz, ^{3}J(C4,P) = 3.3 Hz, ^{3}J(C6,P) = 3.3 Hz, ^{2}J(Ad2,P) = 7.3 Hz, ^{1}J(Ad1,P) = 4.6 Hz, ^{3}J(Ad3,P) = 9.9 Hz, ^{3}J(C1,Ag) = 2.3 Hz, ^{3}J(C3,Ag) = 5.9 Hz, ^{2}J(C2,Ag) = 2.6 Hz, ^{4}J(C4,Ag) = 1.4 Hz, ^{3}J(Ad2,Ag) = 1.9 Hz, ^{2}J(Ad1,Ag) = 4.3 Hz.

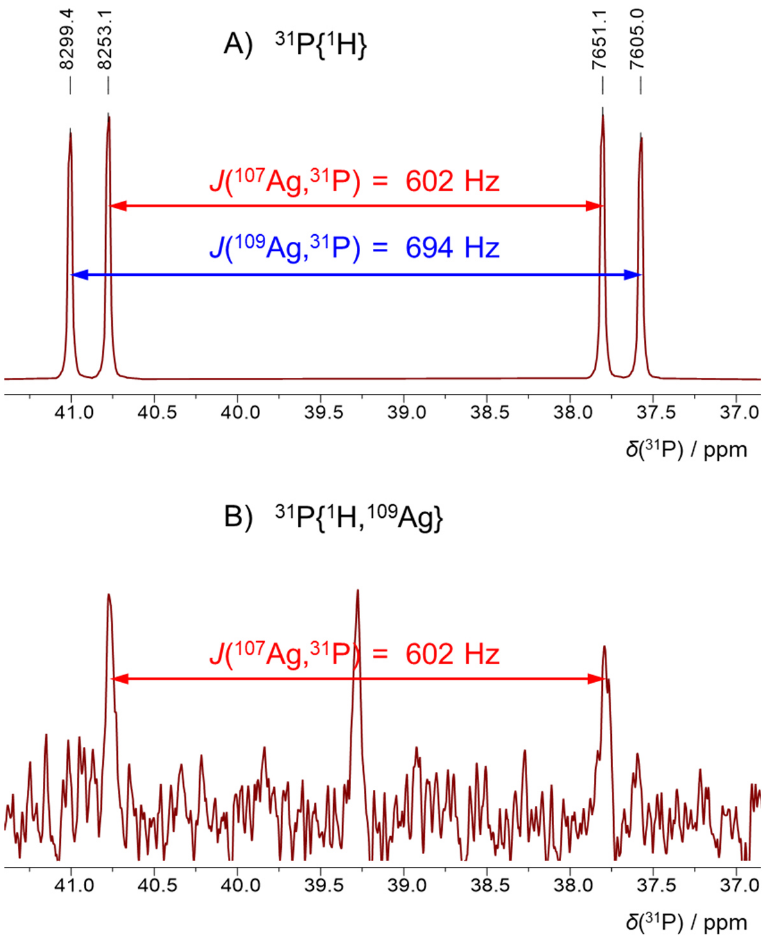

Figure S7. A) ^{31}P NMR spectrum with proton decoupling and B) ^{31}P NMR spectrum with mutual proton and ^{109}Ag decoupling (irradiation offset 650 ppm) of **1AgCl** in CD_2Cl_2 at 25 °C.

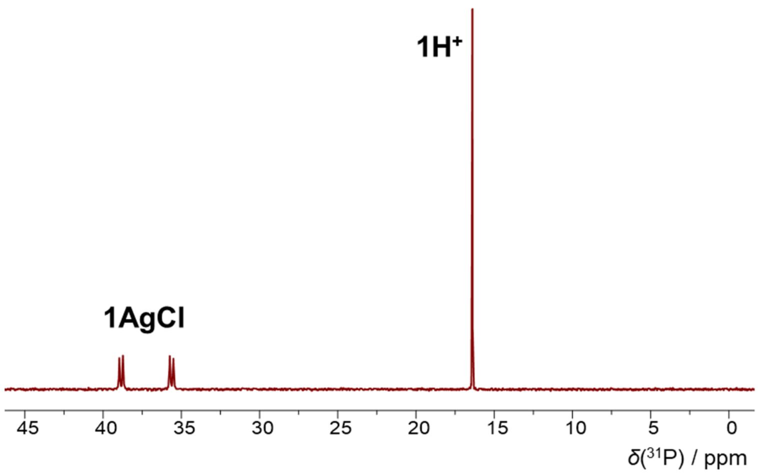

Figure S8 . ^{31}P NMR spectrum with proton decoupling of **1AgCl** after addition of triflic acid in CD_2Cl_2 at −70 °C.

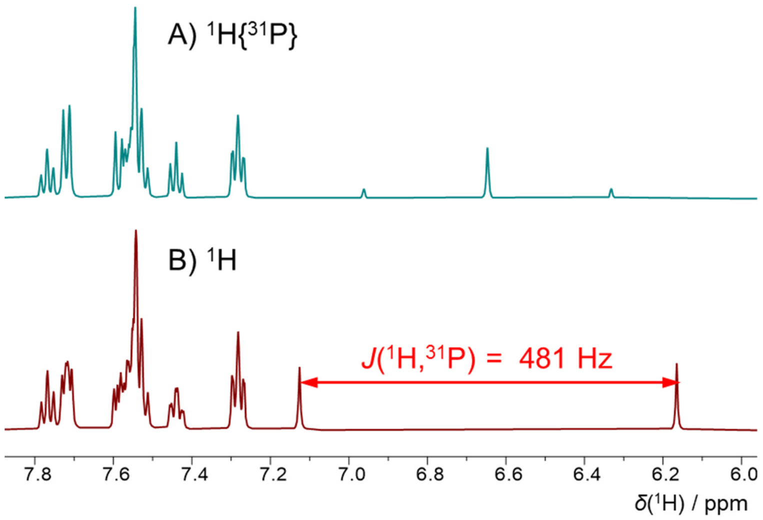

Figure S9. The aromatic and PH region of ^1H NMR spectrum of **1AgCl** after addition of triflic acid in CD_2Cl_2 at −70 °C with A) ^{31}P decoupling and B) without decoupling. This experiment confirms that the two peaks centered around 6.65 ppm form a doublet and correspond to a hydrogen atom directly attached to phosphorus with a large one-bond coupling of 481 Hz.

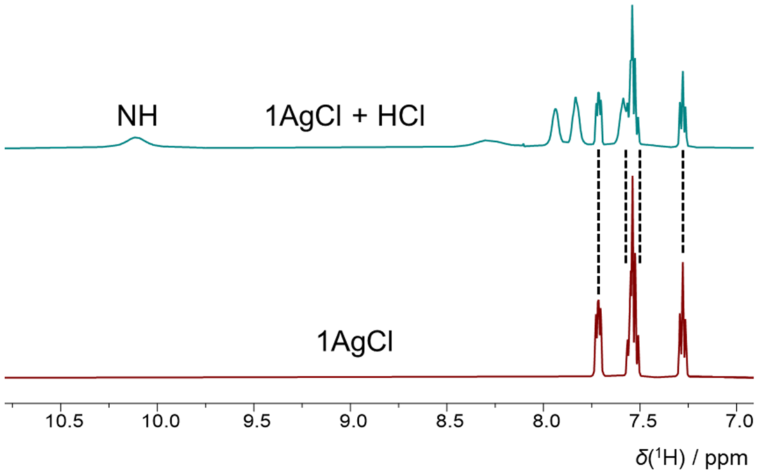

Figure S10. The aromatic and NH region of ^1H NMR spectrum of **1AgCl** before and after addition of HCl in CD_2Cl_2 at −70 °C.

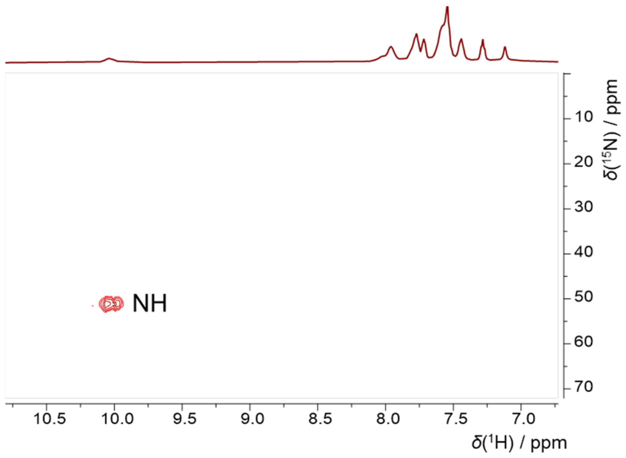

Figure S11. The aromatic and NH region of ¹H,¹⁵N-HSQC spectrum of **1AgCl** after addition of HCl in CD_2Cl_2 at −70 °C. The observation of the cross-peak confirms that the signal at 10.1 ppm corresponds to an N–H hydrogen atom.

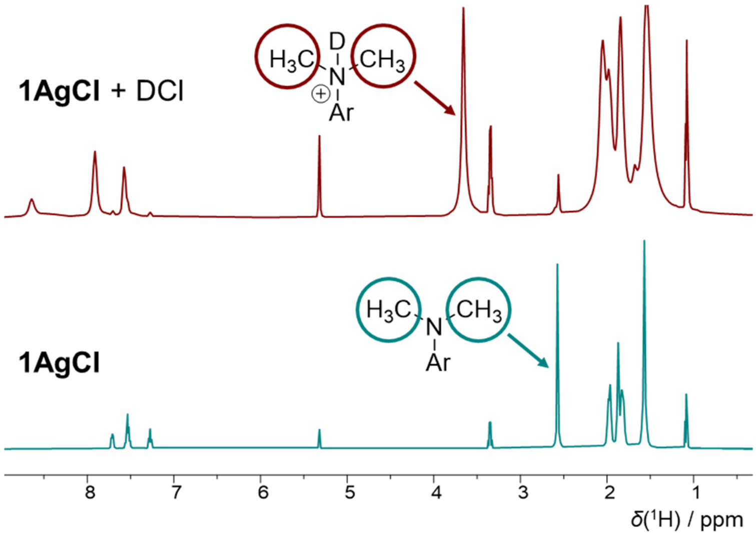

Figure S12. The ¹H NMR spectrum of **1AgCl** before and after addition of DCl in CD_2Cl_2 at −70 °C. The most remarkable change is the shift of the dimethylamino signal from 2.57 to 3.65 ppm.

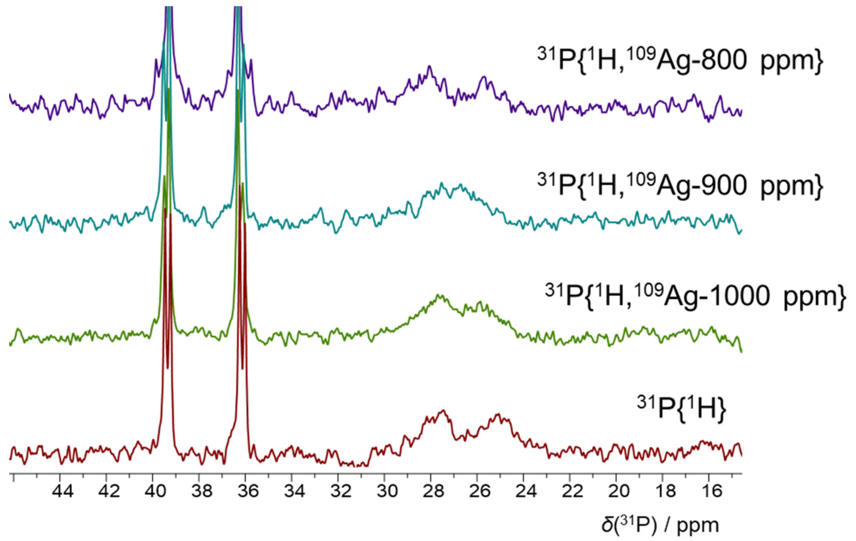

Figure S13. The ^{31}P NMR spectra of **1AgCl** after addition of HCl in dioxane with mutual proton and ^{109}Ag decoupling as a function of ^{109}Ag decoupling frequency (top 3 traces). ^{31}P NMR spectrum with only hydrogen decoupling (bottom trace). The left peaks correspond to unreacted **1AgCl**, whereas the right peaks were assigned to **1AgCl₂H** complex.

Calculated equilibrium geometries

Table S1. Equilibrium geometries of **$1AgCl_2H_{Cl}$** and **$1AgCl_2H_{Ag}$** at various level of theory (incl. different implicit solvents, various starting geometries using and MP2 or PBE0-D3/def2TZVPP/PCM methods). The results illustrate that **$1AgCl_2H_{Ag}$** is stabilized in more polar conditions.

PBE0	Start from:		ε=0, gas phase	ε=9, in DMC	ε=80, in water
	**$1AgCl_2H_{Cl}$**	H...Ag	2.597	2.575	2.538
		H...Cl	1.951	2.126	2.231
		E(PBE0, a.u.)	-2554.02473	-2554.06215	-2554.07167
	**$1AgCl_2H_{Ag}$**	H...Ag	2.594	2.594	2.285
		H...Cl	1.955	1.955	2.703
		E(PBE0, a.u.)	-2554.02447	-2554.06116	-2554.07196
		**Δ*E* (kcal/mol)**	**0.17**	**0.62**	**-0.19**
MP2			ε=0, gas phase	ε=9, in DMC	ε=80, in water
	**$1AgCl_2H_{Cl}$**	H...Ag	2.547	2.567	2.541
		H...Cl	1.923	2.148	2.241
		E(MP2, a.u.)	-2544.973125	-2545.022595	-2545.034715
	**$1AgCl_2H_{Ag}$**	H...Ag	2.548	2.361	2.343
		H...Cl	1.923	2.536	2.619
		E(MP2, a.u.)	-2544.973145	-2545.022113	-2545.035242
		**Δ*E* (kcal/mol)**	**-0.01**	**0.30**	**-0.33**

Table S2. Energies of the model systems.

	CCSDT/TZ	MONO1/TZ	MONO2/TZ	E_int_TZ	HF/TZ	MONO1/TZ	MONO2/TZ	E_HF_int_TZ
1modelB_AgClH	-1162.922718	-134.9920728	-1027.93	**-0.8**	-1160.51	-134.288	-1026.239921	**10.5**
1modelB_AgClH_GM	-1162.95313	-134.9961264	-1027.94	**-11.3**	-1160.55	-134.295	-1026.249833	**-3.3**
1modelA_AgClH_Plus	-1163.313251	-135.3627892	-1027.93	**-12.8**	-1160.91	-134.666	-1026.239946	**-0.2**
1modelA_AgClH_Plus_GM	-1163.360135	-135.3635642	-1027.94	**-34.5**	-1160.97	-134.668	-1026.252818	**-31.3**
1modelB_AuClH	-1151.621267	-134.9920359	-1016.63	**0.7**	-1149.26	-134.288	-1014.990563	**12.0**
1modelB_AuClH_GM	-1151.645253	-134.9966824	-1016.64	**-6.9**	-1149.29	-134.295	-1014.997634	**-0.7**
1modelA_AuClH_Plus	-1152.0085	-135.3627897	-1016.63	**-9.3**	-1149.65	-134.666	-1014.990583	**4.3**
1modelA_AuClH_Plus_GM	-1152.049215	-135.3637945	-1016.64	**-30.8**	-1149.71	-134.668	-1014.998407	**-27.3**
1modelB_CuClH	-2656.036872	-134.9919299	-2521.04	**-0.7**	-2653.44	-134.288	-2519.165544	**9.8**
1modelB_CuClH_GM	-2656.077982	-134.996717	-2521.04	**-28.0**	-2653.48	-134.294	-2519.161421	**-12.5**
1modelA_CuClH_Plus	-2656.428706	-135.3631851	-2521.04	**-13.2**	-2653.83	-134.667	-2519.165558	**-1.1**
1modelA_CuClH_Plus_GM	-2656.467927	-135.3636797	-2521.05	**-32.3**	-2653.89	-134.668	-2519.177603	**-29.6**
H2O_Dimethylammonium	-211.7505789	-135.3650207	-76.3587	**-16.9**	-210.753	-134.669	-76.06056393	**-14.6**
Benzene_Dimethylammonium	-367.3334592	-135.3656898	-231.941	**-16.9**	-365.467	-134.67	-230.7815808	**-10.1**
Xe_Dimethylammonium	-463.9391411	-135.3645935	-328.568	**-4.1**	-462.97	-134.67	-328.298689	**-1.1**
2modelA_AuClH_Plus								
**1modelA_AgClH_Plus**								
**PBE0/ECP-def2TZVPP**								
Ag		-1164.160319	-135.417	-1028.72	**-15.8**			
Au		-1152.926271	-135.417	-1017.49	**-12.0**			
**PBE0/X2C_SOC_FINNUCL_x2c-TZVPPall-2c**								
Ag		-6335.129305	-135.476	-6199.63	**-15.8**			
Au		-20052.61251	-135.476	-19917.1	**-13.2**			
**2modelA_AuClH_Plus**								
**PBE0/ECP-def2TZVPP**								
Au		-1012.678938	-174.7	-837.953	**-16.1**			
**PBE0/X2C_SOC_FINNUCL_x2c-TZVPPall-2c**								
Au		-19911.6467	-174.774	-19736.8	**-16.8**			

	HF/QZ	MP2_corrQZ	MP2_corrTZ	CCSDT_corr/TZ	**CCSDT/CBS**
1modelB_AgClH	-1160.531994	-2.537263	-2.281266951	-2.411717822	**-1163.39**
1modelB_AgClH_GM	-1160.571151	-2.531168	-2.274079144	-2.403459828	**-1163.42**
1modelA_AgClH_Plus	-1160.927582	-2.52923	-2.274337828	-2.406884866	**-1163.78**
1modelA_AgClH_Plus_GM	-1160.99194	-2.513715	-2.257324397	-2.389778854	**-1163.83**
1modelB_AuClH	-1149.280517	-2.4934	-2.244777538	-2.362132677	**-1152.07**
1modelB_AuClH_GM	-1149.315432	-2.483006	-2.234217413	-2.35172285	**-1152.10**
1modelA_AuClH_Plus	-1149.671343	-2.487216	-2.239744968	-2.35877675	**-1152.46**
1modelA_AuClH_Plus_GM	-1149.731941	-2.466992	-2.217912857	-2.339277552	**-1152.50**
1modelB_CuClH	-2653.459748	-2.874353	-2.559528992	-2.599186797	**-2656.60**
1modelB_CuClH_GM	-2653.497641	-2.879228	-2.56483265	-2.602624531	**-2656.64**
1modelA_CuClH_Plus	-2653.856363	-2.867352	-2.553153027	-2.594635844	**-2656.99**
1modelA_CuClH_Plus_GM	-2653.915337	-2.846884	-2.533560918	-2.575232498	**-2657.03**
H2O_Dimethylammonium	-210.766664	-1.019245	-0.922406041	-0.997629317	**-211.93**
Benzene_Dimethylammonium	-365.4884339	-1.900812	-1.725611061	-1.866199854	**-367.66**
Xe_Dimethylammonium	-462.9791843	-1.069687	-0.892239201	-0.96882949	**-464.25**
2modelA_AuClH_Plus	-1009.013204	-3.159249	-2.834385866	-2.961575793	**-1012.54**

	**MONO1** HF/QZ	MP2_corrQZ	MP2_corrTZ	CCSDT_corr/TZ	**CCSDT/CBS**
1modelB_AgClH	-134.296	-0.70851	-0.64442	-0.70428	**-135.11**
1modelB_AgClH_GM	-134.303	-0.70693	-0.64211	-0.7015	**-135.12**
1modelA_AgClH_Plus	-134.675	-0.69784	-0.63452	-0.69662	**-135.48**
1modelA_AgClH_Plus_GM	-134.676	-0.69792	-0.6338	-0.69584	**-135.48**
1modelB_AuClH	-134.296	-0.70863	-0.64452	-0.70439	**-135.11**
1modelB_AuClH_GM	-134.303	-0.70705	-0.64255	-0.70197	**-135.12**
1modelA_AuClH_Plus	-134.674	-0.69807	-0.63474	-0.69684	**-135.48**
1modelA_AuClH_Plus_GM	-134.676	-0.69784	-0.63373	-0.69578	**-135.48**
1modelB_CuClH	-134.296	-0.70853	-0.64434	-0.70419	**-135.11**
1modelB_CuClH_GM	-134.302	-0.70753	-0.64324	-0.70274	**-135.12**
1modelA_CuClH_Plus	-134.675	-0.69785	-0.63441	-0.6965	**-135.48**
1modelA_CuClH_Plus_GM	-134.676	-0.69784	-0.63368	-0.69572	**-135.48**
H2O_Dimethylammonium	-134.677	-0.69759	-0.63387	-0.69596	**-135.48**
Benzene_Dimethylammonium	-134.678	-0.69765	-0.63408	-0.69612	**-135.48**
Xe_Dimethylammonium	-134.678	-0.69705	-0.63257	-0.69465	**-135.48**
2modelA_AuClH_Plus	-173.728	-0.91412	-0.83217	-0.9131	**-174.78**

	**MONO2** HF/QZ	MP2_corrQZ	MP2_corrTZ	CCSDT_corr/TZ	**CCSDT/CBS**	**E_int_CCSDT/CBS)**	**E_int_MP2_QZ**	**E_int_HF_QZ**	**delta_E_SOC_rel**	**E_int_Final**
1modelB_AgClH	-1026.25	-1.807957147	-1.61688226	-1.68941546	**-1028.27**	-1.7	**-2.6**	**10.5**		
1modelB_AgClH_GM	-1026.26	-1.808867213	-1.617474837	-1.68915284	**-1028.28**	-12.3	**-12.9**	**-3.3**		
1modelA_AgClH_Plus	-1026.25	-1.808324365	-1.617556091	-1.69008178	**-1028.27**	-13.7	**-14.7**	**-0.2**	0	**-13.7**
1modelA_AgClH_Plus_GM	-1026.27	-1.809062958	-1.617406098	-1.68875897	**-1028.29**	-35.2	**-35.5**	**-31.3**		
1modelB_AuClH	-1015	-1.762705652	-1.578999603	-1.63975083	**-1016.96**	-0.2	**-1.9**	**12.0**		
1modelB_AuClH_GM	-1015.01	-1.764772226	-1.580840279	-1.63991071	**-1016.97**	-7.3	**-7.8**	**-0.7**		
1modelA_AuClH_Plus	-1015	-1.76302992	-1.579619901	-1.6403698	**-1016.96**	-10.1	**-12.2**	**4.2**	-1.1	**-11.2**
1modelA_AuClH_Plus_GM	-1015.01	-1.762227496	-1.577876416	-1.63797339	**-1016.97**	-31.4	**-31.6**	**-27.3**		
1modelB_CuClH	-2519.18	-2.147443217	-1.897585618	-1.87820864	**-2521.49**	-1.6	**-1.7**	**9.8**		
1modelB_CuClH_GM	-2519.18	-2.139798089	-1.891085942	-1.87522916	**-2521.48**	-29.5	**-32.6**	**-12.6**		
1modelA_CuClH_Plus	-2519.18	-2.148306052	-1.898306885	-1.8789192	**-2521.49**	-14.1	**-14.5**	**-1.2**	0	**-14.1**
1modelA_CuClH_Plus_GM	-2519.19	-2.144245893	-1.89563427	-1.87518643	**-2521.50**	-32.9	**-32.6**	**-29.6**		
H2O_Dimethylammonium	-76.0659	-0.317387603	-0.284682978	-0.29809505	**-76.42**	-17.4	**-17.3**	**-14.7**		
Benzene_Dimethylammonium	-230.794	-1.189473981	-1.078321646	-1.15925895	**-232.15**	-17.4	**-18.7**	**-10.1**		
Xe_Dimethylammonium	-328.299	-0.367280748	-0.254477426	-0.26931315	**-328.76**	-4.3	**-4.4**	**-1.1**		
2modelA_AuClH_Plus	-835.286	-2.217896995	-1.975768721	-2.02569629	**-837.73**	-15.1	**-17.0**	**0.0**	-0.7	**-15.8**

Calculated NMR parameters

All NMR properties correspond to ^{1}H, ^{31}P ^{15}N and ^{109}Ag nuclei. "W" stands for water.

Non-protonated structures

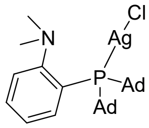

m7
$\delta^{31}P$: 41.8
$\delta^{109}Ag$: 665.8
J_{AgP} -704.6

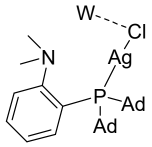

05-06-0-1
$\delta^{31}P$: 42.0
$\delta^{109}Ag$: 665.8
J_{AgP}: -651.5

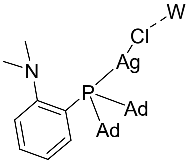

15-01-0-1
$\delta^{31}P$: 41.3
$\delta^{109}Ag$: 675.9
J_{AgP}: -649.7
J_{AgN}: 3.3

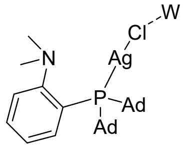

16-07-0-1
$\delta^{31}P$: 39.8
$\delta^{109}Ag$: 678.4
J_{AgP}: -654.1

Protonated structures

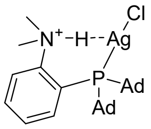

m1
$\delta^{31}P$:34.3
$\delta^{109}Ag$: 710.3
$\delta^{1}H$: 11.2
J_{AgP}: -585.3
J_{NH}: 44.7
J_{AgH}:-5.2

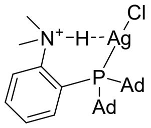

01-04-1-1
$\delta^{31}P$:33.4
$\delta^{109}Ag$: 669.2
$\delta^{1}H$: 10.8
J_{AgP}:-482.6
J_{NH}: 46.4

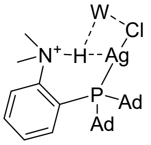

17-09-1-1
$\delta^{31}P$: 36.5
$\delta^{109}Ag$: 620.3
$\delta^{1}H$: 8.9
J_{AgP}:-561
J_{NH}: 49.7

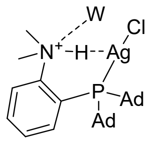

14-03-1-1
$\delta^{31}P$: 36.6
$\delta^{109}Ag$: 637.6
$\delta^{1}H$: 9.9
J_{AgP}: -552
J_{NH}: 48.2

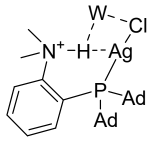

14-05-1-1
$\delta^{31}P$: 34.7
$\delta^{109}Ag$: 626.4
$\delta^{1}H$: 10.4
J_{AgP}: -519
J_{NH}: 46.7

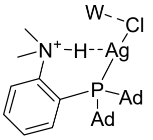

14-09-1-1
$\delta^{31}P$: 36.3
$\delta^{109}Ag$:621.3
$\delta^{1}H$: 9.3
J_{AgP}: -555.0
J_{NH}: 49.12

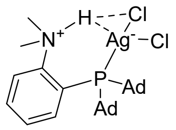

m2
$\delta^{31}P$: 29.0
$\delta^{109}Ag$: 924.6
$\delta^{1}H$: 12.0
J_{AgP}: -425.6
J_{NH}: 45.2

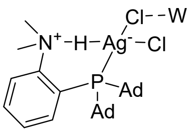

06-09-0-1
$\delta^{31}P$: 29.2
$\delta^{109}Ag$: 973.9
$\delta^{1}H$: 11.2
J_{AgP}: -483.6
J_{NH}: 48.4

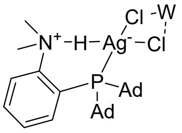

08-06-0-1
$\delta^{31}P$: 27.7
$\delta^{109}Ag$: 969.1
$\delta^{1}H$: 11.5
J_{AgP}: -494.0
J_{NH}: 47.8

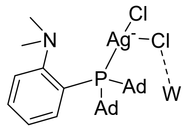

07-04-1-1
$\delta^{31}P$: 25.7
$\delta^{109}Ag$: 926.6
J_{AgP}: -555.2

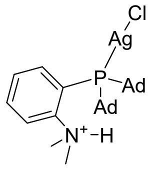

m5
$\delta^{31}P$: 103.8
$\delta^{10x}Ag$: 555.5
$\delta^{1}H$: 6.9
J_{AgP} -692
J_{NH}: 48.7

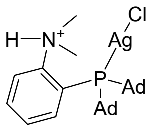

m6 7.4+
$\delta^{31}P$: 51.7
$\delta^{10x}Ag$: 661.8
$\delta^{1}H$: 6.6
J_{AgP} -752
J_{NH}: 47.8

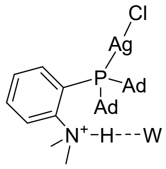

17-05-1-1
$\delta^{31}P$: 119.3
$\delta^{10x}Ag$: 568.5
$\delta^{1}H$: 9.4
J_{AgP} : -612
J_{NH}: 50.5

Structures without silver

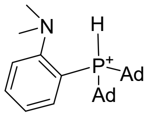

m4
$\delta^{31}P$: 29.8
$\delta^{1}H$: 6.53
J_{PH}:419.8

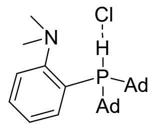

m9
$\delta^{31}P$: 10.8
$\delta^{1}H$: 15.8
J_{PH}:443.2

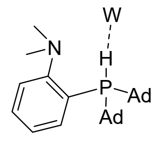

m11
$\delta^{31}P$: 31.5
$\delta^{1}H$: 8.0
J_{PH}:431.2

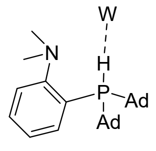

09-07-1-1
$\delta^{31}P$: 19.2
$\delta^{1}H$: 7.8
J_{PH}:442.3

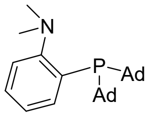

m8
$\delta^{31}P$: 25.5

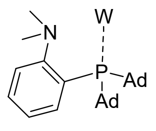

10-04-0-1
$\delta^{31}P$: 16.3

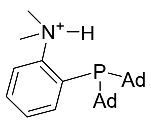

m3
$\delta^{31}P$: -0.7
$\delta^{1}H$: 12.4
J_{NH}: 44.8

Figure S14. Calculated NMR parameters for various additional systems considered in the study.

Bonding Analyses

EDA and NOECV Analyses. To this aim, we also carried out energy decomposition analysis (EDA)[S1] which enables to understand interactions between molecular fragments in molecules. To better understand the M•••H interactions we run EDA on model systems $\mathbf{1^{modelA}MClH^{+}}$. It is worth noting that the two fragments chosen for energy decomposition were MCl and 1H+, which is an approximation that does not account for the clean M•••H interaction; however, there is no other viable choice. EDA generally decomposes the interaction between two subsystems into Pauli repulsion, electrostatic, orbital and dispersion terms. The computed data for the $\mathbf{1M^{modelA}ClH^{+}}$ series are listed in Table S3. The *orbital interaction* part of EDA can be further decomposed into interatomic "channels" between fragments of the system using Natural Orbitals For Chemical Valence (NOCV) analysis.[S2] Among the produced NOCVs we have found a single localized NOCV corresponding to *M*•••H interaction in each of the $\mathbf{1M^{modelA}ClH^{+}}$. Figure S15 shows this NOCV for $\mathbf{1^{modelA}AgClH^{+}}$ and $\mathbf{2^{modelA}AuClH^{+}}$ for comparison.

The NOCV for the interacting M•••H unit along $\mathbf{1^{modelA}ClH^{+}}$ series give orbital interaction energies of −11.2, −11.4, and −16.4 kcal mol^{-1} for Cu, Ag, and Au, respectively. This mirrors the delocalization indices of the MH interactions from the QTAIM analysis, which were 0.12, 0.13, and 0.20, respectively. However, the steric interaction term is ~ 7 kcal mol^{-1} more positive in the case of Au complex, which destabilizes the overall interaction compared to its Ag and Cu counterparts. This might explain why we have observed smaller interaction energies for the Au models compared to Cu and Ag models, despite all bonding analyses indicating stronger M•••H interaction in the former case.

Table S3. EDA decomposition[a,b] and NOCV for model systems $\mathbf{^{M}1\mathit{M}ClH^{+}}$ and for system $\mathbf{^{M}2AuClH^{+}}$.

System	E_{bonding}	$E_{\text{dispersion}}$	$E_{\text{orbital interactions}}$	$E_{\text{NOCV,MH}}$	$E_{\text{NOCV,rest}}$	E_{steric}	$E_{\text{Pauli repulsion}}$	$E_{\text{electrostatic}}$	$\text{Eig}_{\text{NOCV,MH}}$[c]
^{M}**1CuClH**$^{+}$	−17.01	−4.65	−22.83	−11.2	−11.4	10.48	25.11	−14.63	−0.27
^{M}**1AgClH**$^{+}$	−15.48	−4.49	−22.83	−11.4	−11.6	11.84	25.28	−13.44	−0.27
^{M}**1AuClH**$^{+}$	−12.10	−4.24	−26.76	−16.4	−10.3	18.90	30.14	−11.24	−0.32
^{M}**2AuClH**$^{+}$	−15.88	−5.44	−20.69	−10.6	−10.1	10.25	26.22	−15.97	−0.26

[a] The energy decompositions are characterized by the following energy relations: $E_{\text{steric}} = E_{\text{Pauli}} + E_{\text{electrost.}}$; $E_{\text{bonding}} = E_{\text{dispersion}} + E_{\text{orbital interactions}} + E_{\text{steric}}$; $E_{\text{steric}} = E_{\text{Pauli repulsion}} + E_{\text{electrost.}}$; $E_{\text{orbital interactions}} = E_{\text{NOCV,MH}} + E_{\text{NOCV,rest}}$

[b] All energies are in kcal mol^{-1}.

[c] Eigenvalue of NOCV corresponding to the M-H interaction.

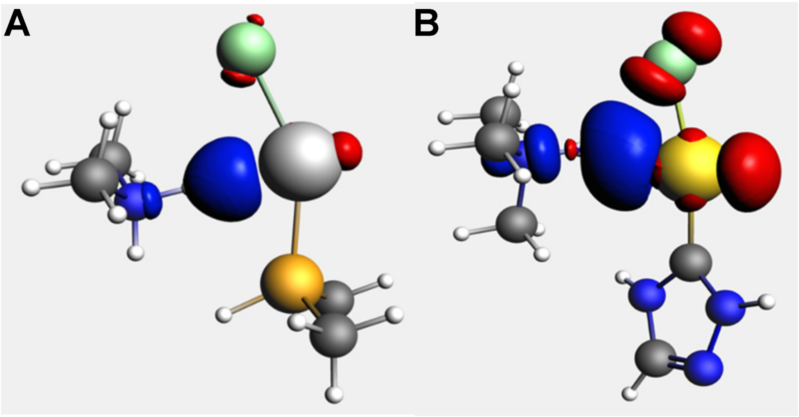

Figure S15. The M•••H interacting NOCV in $1^{modelA}AgClH^{+}$ and $2^{modelA}AuClH^{+}$.

Close N-H•••Ag contacts from Cambridge Structural Database (CSD)

We conducted a search of the CSD with ConQuest program 2022.3.0 (CSD version 5.43, with updates up to November 2022) for possible examples of Ag•••HN interactions. We limited the search to complexes containing with Ag-H distances 0-2.5 Å, Ag-H-N angles 120–180°, and Ag-N distances 2.5-10 Å. Several structures fulfilling these criteria were identified (Table S4), suggesting that short Ag•••HN contacts may have been observed crystallographically. However, neutron diffraction would be needed to ascertain the position of the H atoms in these structures.

Table S4. Close Ag•••H-N contacts in the CSD.[a]

ID	d(Ag-H) [Å]	α (Ag-H-N) [°]	d(Ag-N) [Å]	Ref.
HABBOF	2.367	123.052	2.941	S3
JOXZOO	2.494	123.569	3.070	S4
OLIVOW	2.379	142.249	3.103	S5
OLIVOW	2.140	139.563	2.850	S5
QAKRIE	2.483	134.349	3.144	S6
QAKSAX	2.493	135.832	3.163	S6
SASKUV	2.395	122.102	2.972	S7
TILGEB	2.494	133.025	3.177	S8
XOFROB	2.291	133.998	2.956	S9
XOFROB	2.126	143.710	2.873	S9

[a] CSD search criteria: Ag-H distance 0-2.5 Å, Ag-H-N angle 120–180°, and Ag-N distance 2.5-10 Å. One additional entry, DEZGUL,[S10] was an obvious mistake, because the X-ray structure features doubly protonated pyrimidines with no counterions.

References

(S1) Bickelhaupt, F. M.; Baerends, E. J. Kohn-Sham Density Functional Theory: Predicting and Understanding Chemistry. *Rev. Comput. Chem.* **2000,** 1-86. doi:10.1002/9780470125922.ch1

(S2) Mitoraj, M.; Michalak, A. Donor–Acceptor Properties of Ligands from the Natural Orbitals for Chemical Valence. *Organometallics* **2007,** *26,* 6576-6580. doi:10.1021/om700754n

(S3) Zovko, C.; Bestgen, S.; Schoo, C.; Görner, A.; Goicoechea, J. M.; Roesky, P. W. A Phosphine Functionalized β-Diketimine Ligand for the Synthesis of Manifold Metal Complexes. *Chem. - Eur. J.* **2020,** *26,* 13191-13202. doi:10.1002/chem.202001357

(S4) Meng, P.; Brock, A.; Xu, Y.; Han, C.; Chen, S.; Yan, C.; McMurtrie, J.; Xu, J. Crystal Transformation from the Incorporation of Coordinate Bonds into a Hydrogen-Bonded Network Yields Robust Free-Standing Supramolecular Membranes. *J. Am. Chem. Soc.* **2020,** *142,* 479-486. doi:10.1021/jacs.9b11336

(S5) Zhu, P.; Sun, L.; Sheng, N.; Sha, J.; Liu, G.; Yu, L.; Qiu, H.; Li, S. Tuning the Helical Structures of Wells–Dawson Polyoxometalate Based Hybrid Compounds by Using Isomeric Ligands. *Crystal Growth & Design* **2016,** *16,* 3215-3223. doi:10.1021/acs.cgd.6b00119

(S6) Hu, H.; Yeh, C.; Chen, J. Synthesis and Structural Characterisation of Ag^{I} Complexes with *N*,*N'*-Bis(2-pyridyl)oxalamide and the Anion of *N*-(2-Pyridyl)oxalamic Acid. *Eur. J. Inorg. Chem.* **2004,** *2004,* 4696-4701. doi:10.1002/ejic.200400101

(S7) Zhang, J.; Tan, G.; Liu, B.; Dai, Y.; Xu, N.; Wen, W.; Cao, C.; Xiao, H. A family of silver(I) complexes built with 2-sulfoterephthalic acid monosodium salt and different aminopyridine ligands: Syntheses, structures and properties. *J. Mol. Struct.* **2017,** *1136,* 196-203. doi:10.1016/j.molstruc.2017.02.011

(S8) Chen, X.; Wang, S.; Bai, J.; Li, Y. A 3-D open network constructed from metallamacrocycle chains with a simple 'corner' ligand of 2,4-diamino-6-methyl-1,3,5-triazine(dmt) via cross-linked hydrogen bonds. *J. Coord. Chem.* **2007,** *60,* 1941-1947. doi:10.1080/00958970601183466

(S9) Li, M.; Sha, J.; Zong, X.; Sun, J.; Yan, P.; Li, L.; Yang, X. Assembly of Polyoxometalate-Based Hybrids with Different Helical Channels upon Subtle Ligand Variation. *Crystal Growth & Design* **2014,** *14,* 2794-2802. doi:10.1021/cg500045q

(S10) Lee, K.; Chen, J. C. C.; Huang, C.; Lin, I. J. B. Rectangular architectures formed by acyclic diamido-metal-N-heterocyclic carbenes with skewed conformation. *CrystEngComm* **2007,** *9,* 278. doi:10.1039/b702032p
